# Magnetic nanochain integrated microfluidic biochips

**DOI:** 10.1038/s41467-018-04172-1

**Published:** 2018-05-01

**Authors:** Qirong Xiong, Chun Yee Lim, Jinghua Ren, Jiajing Zhou, Kanyi Pu, Mary B. Chan-Park, Hui Mao, Yee Cheong Lam, Hongwei Duan

**Affiliations:** 1 School of Chemical and Biomedical Engineering, Nanyang Technological University, 70 Nanyang Drive, Singapore, 637457 Singapore; 2 School of Mechanical and Aerospace Engineering, Nanyang Technological University, 50 Nanyang Avenue, Singapore, 639798 Singapore; 3 Cancer Center, Union Hospital, Huazhong University of Science & Technology, 430022 Wuhan, China; 4 Department of Radiology & Imaging Science, School of Medicine, Emory University, Atlanta, GA 30322 USA

## Abstract

Microfluidic biochips hold great potential for liquid analysis in biomedical research and clinical diagnosis. However, the lack of integrated on-chip liquid mixing, bioseparation and signal transduction presents a major challenge in achieving rapid, ultrasensitive bioanalysis in simple microfluidic configurations. Here we report magnetic nanochain integrated microfluidic chip built upon the synergistic functions of the nanochains as nanoscale stir bars for rapid liquid mixing and as capturing agents for specific bioseparation. The use of magnetic nanochains enables a simple planar design of the microchip consisting of flat channels free of common built-in components, such as liquid mixers and surface-anchored sensing elements. The microfluidic assay, using surface-enhanced Raman scattering nanoprobes for signal transduction, allows for streamlined parallel analysis of multiple specimens with greatly improved assay kinetics and delivers ultrasensitive identification and quantification of a panel of cancer protein biomarkers and bacterial species in 1 μl of body fluids within 8 min.

## Abstract (web summary)

Microfluidic platforms are an attractive setup for performing clinical tests but integrated liquid mixing and bioseparation is difficult at small scales. Here Xiong et al. propose magnetic nanochains which can stir the solution and capture agents and thus enable liquid analysis in a short amount of time.

## Introduction

Microfluidic systems that offer precise control of fluids, low sample and reagent consumption, and rapid sample processing are of considerable interest for the development of miniaturized, portable and low-cost analytical platforms^1,2^. In particular, the identification and quantification of molecular and cellular targets using microfluidic biochips are under intense research for a wide spectrum of applications ranging from fundamental biology to clinical diagnostics^3–5^. Rapid, multiplexed detection of a panel of targets is much needed to address the growing demands for dynamic profiling of analytes, timely diagnosis of heterogeneous diseases and high-throughput screening^6–8^.

Current designs of microfluidic biochips commonly contain built-in components such as sensing element-functionalized surfaces and liquid mixers^9,10^. Biofunctionalized surfaces serve to separate and enrich targets of interest from complex fluid samples, which is key to specific detection in subsequent signal transduction^11,12^ On the other hand, spatial confinement in microchannels leads to low Reynolds number fluids under laminar flow, which causes inefficient mixing across the channels mainly controlled by diffusion^13^. Hence, passive or active mixers are introduced to enhance on-chip liquid mixing and mass transfer, which is critical for improving kinetics and sensitivity of the diffusion-limited on-surface assays in microchips^14–16^. However, despite recent success in the laboratory-scale demonstration of microfluidic bioanalysis, these necessary built-in components inevitably increase structural, fabricating, operational, and translational complexity of the chips. It remains challenging to realize integrated liquid mixing, bioseparation, and signal transduction in simple microfluidic configurations.

Here we report a broadly applicable multiplexing microfluidic biochip based on bioconjugated magnetic nanochains (Magchains). In our Magchain-integrated microchip (MiChip), bioconjugated nanochains are actuated by tailored magnetic fields to play dual-functional roles as nanoscale stir bars to promote rapid active liquid mixing and capture agents for bioseparation. Magnetic nanostructures were previously used in microfluidic devices to label biomarkers for magnetic detection or separation^17–19^. However, highly efficient concerted liquid mixing and bioseparation were not performed by magnetic nanostructures for sensing applications. Decoupling these functions traditionally undertaken by on-chip liquid mixers and sensing elements-immobilized surfaces from microfluidic systems enable a simple planar design of the MiChip consisting of flat channels free of built-in components. The MiChip therefore can be broadly adopted for a diverse range of targets and readily refined into multichannel arrays for parallel sample analysis. In this study, we demonstrate that the use of well-dispersed nanochains under continuous mixing overcomes the problem associated with diffusion-limited assay kinetics, giving rise to a rapid turnaround time of <8 min, in contrast to the inefficient target capture at liquid–solid interfaces in conventional designs. The MiChip assay allows rapid, parallel analysis of small volumes (~1 μl) of body fluid specimens, achieving sensitively and selectively quantification, and profiling of cancer protein markers in serum samples from 20 cancer patients and specific bacteria in human saliva.

## Results

### Design of the MiChip assay

**Fig. 1:**
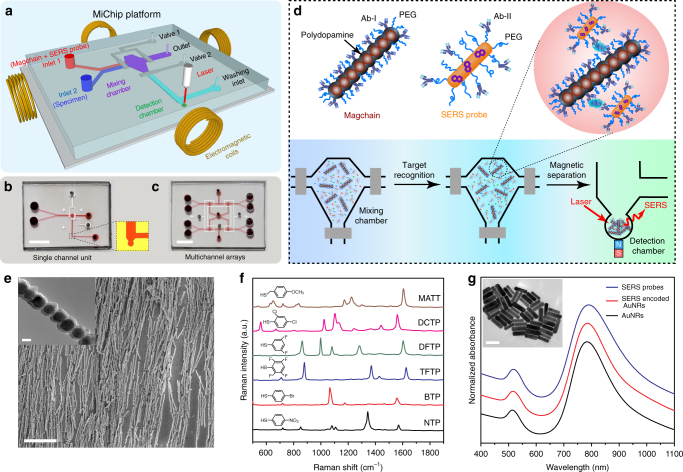
Design of the magnetic nanochain integrated microfluidic chip (MiChip). **a** Schematic illustration of the MiChip assay platform. **b**, **c** Photographs of the MiChip: single channel unit (**b**) and multichannel arrays (**c**). The microchannel was filled with a red dye for better visualization. Scale bar: 0.5 cm. **d** The MiChip assay for the detection of biomarkers. The specimen, antibody-conjugated magnetic nanochains (Magchain) and SERS-encoded probes (SERS probe) are mixed in the mixing chamber. The targets of interest in specimen are recognized by the antibodies on the Magchains and the SERS probes to form sandwich immune complexes. The immune complexes are then isolated into the detection chamber and subjected to Raman spectroscopic detection. **e** SEM image of the magnetic nanochains. Scale bar: 20 µm. Inset: TEM image of magnetic nanochain. Scale bar: 200 nm. **f** SERS spectra of 6 representative SERS-encoded AuNRs. From bottom to top: 4-nitrothiophenol (NTP), 4-bromothiophenol (BTP), 2,3,5,6-tetrafluorothiophenol (TFTP), 3,5-difluorothiophenol (DFTP), 2,4-dichlorothiophenol (DCTP), and 4-methoxy-α-toluenethiol (MATT). **g** UV–vis spectra of original AuNRs, SERS-encoded AuNR, and antibody-conjugated SERS probes. Inset: TEM image of the AuNRs. Scale bar: 100 nm

Figure 1 illustrates the design of the MiChip and the on-chip detection of targets by a sandwich immunoassay based on Magchains and Raman-encoded nanoprobes. As shown in Fig. 1a and b, the basic unit of the polydimethylsiloxane (PDMS)-on-glass MiChip platform features a mixing chamber, a detection chamber, four fluid ports for sample input and waste output, and two pneumatic microvalves that control the fluid delivery into/from the mixing chamber. The dimensions of each part of the chip are shown in Supplementary Fig. 1a. The chambers and channels have a uniform height of 50 μm, with internal surfaces of the MiChip PEGylated to suppress potential biofouling by non-specific constituents in liquid specimens. Of particular note is that the MiChip adopts a simple planar design consisting of flat channels and is free of any target-specific components (Supplementary Fig. 1b). Importantly, the simple design of this basic unit can be easily expanded into integrated multichannel arrays for parallel analysis of multiple specimens. Figure 1c shows a model of a 4-channel system, in which the arrayed units share a common waste outlet and pneumatic microvalves. The individual unit can be operated independently without crosstalk, which is critical for high-throughput screening (see design details in Supplementary Fig. 1c).

Raman spectroscopy was employed in this study for on-chip detection because of its tolerance towards complex, unprocessed liquid samples^20,21^, excellent compatibility with microfluidic devices^22^, its high-spatial resolution, and superior photostability of Raman probes^23,24^. Plasmonic nanostructures with surface-confined electromagnetic field are able to greatly amplify, by an enhancement factor (EF) of 10^5^ or more, the intrinsically weak Raman scattering of molecules in close proximity^25^. Anchoring Raman reporter molecules of different chemical structures on plasmonic nanostructures leads to a library of surface-enhanced Raman scattering (SERS) nanoprobes with the same physical sizes but distinct, sharp spectroscopic signatures that can be excited and differentially detected simultaneously, making them excellent multiplexing tags^26^.

In the MiChip assay, antibody-conjugated magnetic nanochains (Magchain, Fig. 1d) are directed by the external magnetic field to concurrently drive sample mixing and target capture/enrichment. SERS-encoded nanoprobes (SERS probe, Fig. 1d) serve as ultrasensitive signaling probes to realize multiplexed detection. The MiChip assay is streamlined into three key steps (Fig. 1d): First, unprocessed liquid specimen (1.0 μl) and a mixture of Magchains and SERS nanoprobes in PBS buffer (1.0 μl) are injected into the chip via the two inlets, respectively. Second, the liquid delivered into the mixing chamber is confined inside by the pneumatic microvalves 1 and 2, and undergoes mixing driven by the synchronized rotation of the Magchains in a spinning magnetic field generated by four orthogonal electromagnetic coils. During this process, antigen–antibody recognition results in sandwich immune complexes of the Magchains, the targets of interest and the SERS probes in the chamber. Third, microvalve 2 is opened and all the Magchains including those in the immune complex are magnetically collected from the mixing chamber into the detection chamber for Raman spectroscopic detection. The volume difference of mixing and detection chambers leads to an enrichment factor of 100.

To prepare Magchains (Supplementary Fig. 2), superparamagnetic Fe_3_O_4_ nanoparticles of 250 nm diameter (Supplementary Fig. 3) dispersed in dopamine solution of bicine buffer (pH 8.5) are placed in a homogeneous magnetic field (0.05 T) to form linear arrays^27^. Self-polymerization of dopamine at a weak basic condition leads to highly crosslinked, rigid polydopamine that shows strong adhesion against almost any solid substrate^28^. As a result, the adhesive polydopamine forms a conformal coating on the aligned nanoparticles, leading to crosslinked superparamagnetic nanochains (Fig. 1e) with a saturation magnetization of 41.7 emu g^−1^ (Supplementary Fig. 4). Importantly, polydopamine not only serves as the scaffold to lock the nanochain structure, but also allows for convenient surface functionalization by means of spontaneous Michael addition and/or Schiff base reaction (Supplementary Fig. 5) with nucleophilic thiol and amine groups^29^. As such, the nanochains can be sequentially functionalized by target-specific capture antibody (Ab-I) and thiolated poly(ethylene glycol) (PEG), which is introduced to minimize non-specific biofouling in complex body fluids.

Au nanorods (AuNRs) with longitudinal localized surface plasmon resonance (LSPR) centered at 795 nm (Supplementary Fig. 6) were used as the substrates for SERS-encoded nanoprobes to achieve efficient excitation and sensitive detection at near-infrared (NIR) wavelengths (i.e., 785 nm), where the absorption and scattering by complex fluids such as blood is minimal^30^. Raman molecules of distinct vibrational profiles were encoded on the same AuNRs via Au-sulfur bond, followed by surface passivation using thiolated PEG and conjugation of the detection antibody (Ab-II) of the immunoassay (Supplementary Fig. 7). The resultant SERS nanoprobes have the same size but molecule-defined spectral signatures that can be excited at a single wavelength, detected simultaneously, and easily distinguished from each other (Fig. 1f; Supplementary Fig. 8), offering unique advantages for multiplexed detection. LSPR peaks of plasmonic nanostructures are highly sensitive to interparticle distances, experiencing significant red-shifts upon aggregation. UV–vis (Fig. 1g) and Raman spectra (Supplementary Fig. 9) reveal that the three steps of surface functionalization of the AuNRs (length/width = 100/28 nm; LSPR ~795 nm) did not cause significant changes in their LSPR peaks and SERS profiles, indicative of the presence of stable individual AuNRs (Supplementary Fig. 10), which is crucial for their utilization in quantitative analysis.

### Liquid mixing by magnetic nanochains

**Fig. 2:**
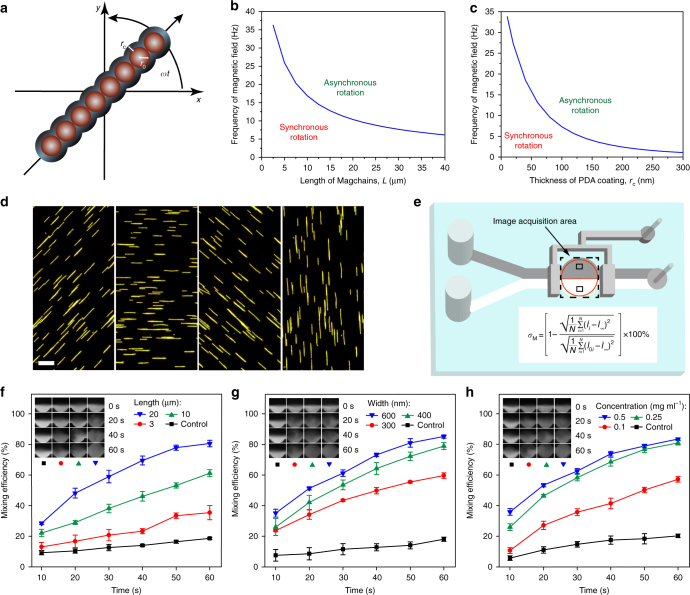
Mixing enhancement by magnetic nanochains. **a** Force analysis of a magnetic nanochain in a rotating external magnetic field. *r*_0_ and *r*_c_ are the radius of magnetic nanoparticles and the thickness of polydopamine (PDA) layer, respectively. **b**, **c** Theoretical analysis results of maximum frequency (*f*_max_) to maintain synchronous rotation of the magnetic nanochains with different length (**b**) and PDA thickness (**c**). **d** Dark-field microscope images of magnetic nanochains responding to an alternating magnetic field, captured by a CCD camera. Scale bar: 20 µm. **e** Schematic of microfluidic chip for the evaluation of mixing efficiency. Inset: equation for evaluation of the mixing efficiency based on fluorescence distribution. **f**–**h** Mixing enhancement by using magnetic chains of different lengths (**f**), widths (**g**), and concentrations (**h**). The insets are the respective images of acquisition area at 0, 20, 40, and 60 s. Error bars indicate the standard deviation of three measurements

The magnetic chains immediately align in a magnetic field and undergo synchronous rotation in response to a rotating magnetic field, offering the possibility of using them as nanoscale stir bars^31,32^. When a rotating magnetic field is applied, two opposing angular torques (i.e., magnetic and viscous torque) act on the magnetic nanochains (Fig. 2a). For the nanochains to maintain a constant angular velocity, the opposing torques need to be balanced at the center of the chain^33^. The maximum frequency (*f*_max_) of the applied alternating field for synchronous rotation of the nanochains is shown as follows (see Supplementary Note 1 for calculation details):

1 $$f_{\max } = \frac{{\chi ^2H_0^2r_0\ln (L/4r_0)}}{{8\pi L\eta \mu _0}}\left( {\frac{{r_0}}{{r_0 + r_{\mathrm{c}}}}} \right)^3,$$

where *χ* is the magnetic susceptibility, *H*_0_ is the strength of applied magnetic field, *η* is the viscosity of the fluid, *μ*_0_ is the permeability constant, *r*_0_ is the radius of the magnetic nanoparticles, *r*_c_ is the thickness of polydopamine coating, and *L* is the length of magnetic nanochain. The parameters of *χ*, *H*_0_, *η*, *μ*_0_, and *r*_0_ are constant in this experimental condition. Based on Eq. 1, it can be observed that *f*_max_ increases with decreasing chain length and thickness of the polydopamine coating (Fig. 2b, c). This means that higher rotation speed can be achieved with shorter chain and thinner coating. On the contrary, longer and thicker chains displace more liquid as they rotate and generate more pronounced convection to promote mixing. Hence, the chain length and thickness need to be optimized to achieve the highest mixing efficiency by seeking a balance between the chain size and rotation speed.

Dark-field imaging (Fig. 2d; Supplementary Movie 1) shows the magnetic nanochains immediately aligned when placed in a homogeneous magnetic field (0.008 T), and took synchronous, localized rotation when two pairs of orthogonal electromagnetic coils were activated periodically. The time-lapse high-speed camera observation (Supplementary Fig. 11; Supplementary Movie 2) shows that the nanochains (length: 20 µm, width: 400 nm) can achieve a maximum synchronous rotation speed of 540 rpm, which is consistent with the speed (10.4 Hz or 624 rpm) estimated in theoretical analysis. Beyond that, the driving magnetic torque generated by the field is insufficient to balance the opposing viscous drag, which increases linearly with the angular velocity. We found that the average length and width of the nanochains can be tailored in the range of 3–20 μm and 300–600 nm, respectively, by controlling reaction conditions such as the reaction time in the magnetic field and the starting concentration of dopamine (Methods and Supplementary Fig. 12).

We evaluated the efficiency of fluid mixing by the rotating magnetic nanochains in a simple Y-shaped microfluidic chip, in which two channels of fluids stream into a circular mixing chamber (300 µm in diameter) that can be closed by two pneumatic microvalves (Fig. 2e). Magnetic nanochains were injected via two separate inlets into the mixing chamber at a flow rate of 50 µl h^−1^, with one of the fluids highlighted by CdSe/ZnS quantum dots (QDs) with emission centered at 550 nm (Supplementary Fig. 13). The QDs serve as a fluorescent marker to quantify the mixing efficiency (*σ*_M_) between the two streams by tracking the fluorescence distribution in the closed mixing chamber. Quantitatively, *σ*_M_ of 0 and 100% represents no mixing and a complete mixing, respectively (Supplementary Note 2)^16^. Time-lapse observation shows that *σ*_M_ is dependent on both structural parameters (length and width) and concentration of the nanochains (Fig. 2f–h; Supplementary Movie 3). For example, in a magnetic field with an alternating frequency of 5 Hz (Supplementary Fig. 14), nanochains with an average length of 20 µm and 3 µm led to *σ*_M_ of 81 and 35%, respectively, after 1 min of mixing (Fig. 2f). In clear contrast, magnetic nanoparticles of the same concentration only led to 21% of mixing (Supplementary Fig. 14), similar to the same sample in absence of the spinning magnetic field (Fig. 2f), underlining the importance of liquid mixing by the nanoscale stir bars for microfluidic assays. Notably, although *σ*_M_ improves with increasing chain width and concentration, it shows marginal improvements after the width and concentration reach 400 nm and 0.25 mg ml^−1^ (Fig. 2g,h), respectively._._ Therefore, magnetic chains of 20 µm in length and 400 nm in width at a concentration of 0.25 mg ml^−1^ was eventually chosen for optimized mixing in the MiChip assay.

### Multiplexed detection of protein biomarkers

**Fig. 3:**
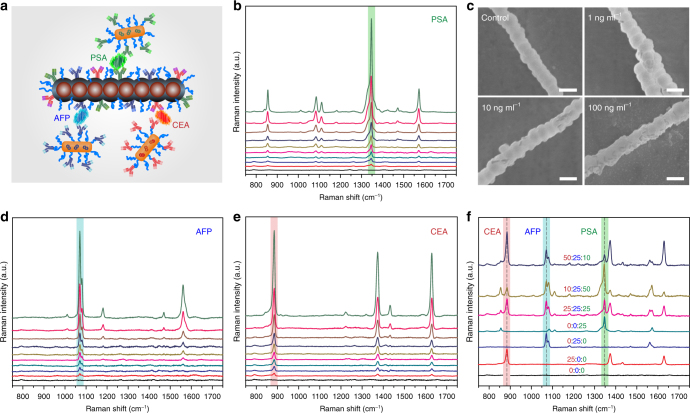
Multiplexed detection of cancer biomarkers on the MiChip platform. **a** Conceptual illustration of the simultaneous detection of PSA (green), AFP (blue), and CEA (red). **b** SERS spectra of different concentrations of PSA in PBS (ranging from 0 to 100 ng ml^−1^). **c** SEM images of Magchain-PSA-SERS probes complex formed at varying concentrations of PSA. Scale bar: 500 nm. **d**, **e** SERS spectra of AFP and CEA at varying concentrations (ranging from 0 to 100 ng ml^−1^). **f** SERS responses to mixtures of the three cancer biomarkers of various ratios

In the first proof-of-concept application, we explored the use of the MiChip platform for multiplexed detection of three serum protein biomarkers: prostate-specific antigen (PSA), carcinoembryonic antigen (CEA), and *α*-fetoprotein (AFP), which are commonly used clinical biomarkers for prostate, colorectal, and hepatocellular cancers, respectively^34,35^. Technologies that allow for sensitive, timely measurement of multiple biomarkers in small volume of specimens hold great promise for screening, early detection, and outcome analysis of complex, heterogeneous diseases such as cancer. Respective capture antibody on the Magchains and detection antibody on the SERS probes for the targets were identified to minimize cross-reactivity. The number of antibodies per Magchain and SERS probe are 6.4 × 10^5^ and 3.2 × 10^2^, respectively, which provide sufficient binding sites for targeted biomarkers in a broad dynamic range based on the use of 4.5 × 10^4^ Magchains and 2.1 × 10^8^ SERS probes in each test. The Magchains for multiplexed assays are modified to carry a nearly equal number of all three capture antibodies (Fig. 3a). Off-chip enzyme-linked immunosorbent assay (ELISA) quantification shows that the Magchains are able to capture 95% of PSA, 90% of AFP, and 91% CEA from standard biomarker solutions of 100 ng ml^−1^, which dropped slightly to 85-88% for the multiplexed Magchains (Supplementary Fig. 15). The capture efficiency of Magchains against mismatched targets is consistently lower than 5%. The high recovery rate and negligible crosstalk of Magchain-based bioseparation are key prerequisites to realize multiplexed assays.

We selected Raman reporters, i.e., 4-nitrothiophenol (NTP), 4-bromothiophenol (BTP), and 2,3,5,6-tetrafluorothiophenol (TFTP) to encode PSA, AFP, and CEA, respectively. ELISA quantification confirms that 5 min of mixing captures 80% of PSA (100 ng ml^−1^) (Supplementary Fig. 16). All of the Magchains can be collected into the detection chamber within 1.5 min (Supplementary Movie 4) by a neodymium magnet (10 × 10 × 5 mm) that leads to a magnetic flux density of about 300 G at the mixing chamber and a spatial magnetic field gradient of about 400 G cm^−1^. Therefore, the streamlined assay is set to complete within 8 min, fractioned into 5 min of Magchain-driven mixing, 1.5 min for magnetic separation, and 1.5 min for liquid transport and spectroscopic readout. The results of the MiChip assay with PSA standards show that in situ reading of SERS intensity at 1341 cm^−1^ of NTP is linearly proportional to PSA concentration in the range of 0.1–100 ng ml^−1^ (Fig. 3b; Supplementary Fig. 17a). The relative standard deviation of the SERS signal remains below 6% in the entire concentration range, indicating good statistical accuracy. The MiChip design allows for easy recovery of separated targets via the detection chamber. As revealed by scanning electron microscopy (SEM) observation, more AuNRs are found on the nanochains (Fig. 3c) recovered from the standard samples with higher PSA concentrations, in line with the SERS results. The control sample without PSA, on the other hand, showed no AuNR on the nanochain surfaces, confirming the high specificity of our design. Also important is that SERS measurements in various control media showed minimal non-specific binding (Supplementary Fig. 18). Besides the sample volume (~1 μl) defined by the capacity of the mixing chamber, we optimized and fixed all the assay conditions in sample (Magchains and SERS probes) loading, magnetic mixing and separation, and spectral acquisition to achieve comparable quantitative analysis. Our results have shown that 10 consecutive measurements gave rise to nearly identical results, confirming excellent photostability of the SERS probes for quantification (Supplementary Fig. 19). Indeed, AFP (Fig. 3d; Supplementary Fig. 17b) and CEA (Fig. 3e; Supplementary Fig. 17c) measurements showed similar trends of linear signal-concentration dependence. Detection limit of the on-chip measurement is ~10 pg ml^−1^ (insets in Supplementary Fig. 17), which is two orders of magnitude lower than that (~1 ng ml^−1^) of ELISA (Supplementary Fig. 20). The well-resolved peaks at 1341, 1071, and 885 cm^−1^ of the three SERS probes allow for readily simultaneous detection of their encoded biomarkers. The number of Raman molecules on AuNRs was controlled to afford similar SERS intensity for the same concentration of biomarkers, which enabled straightforward multiplexed quantification of the biomarkers in a series of samples containing varying concentrations of the target(s) (Fig. 3f).

**Fig. 4:**
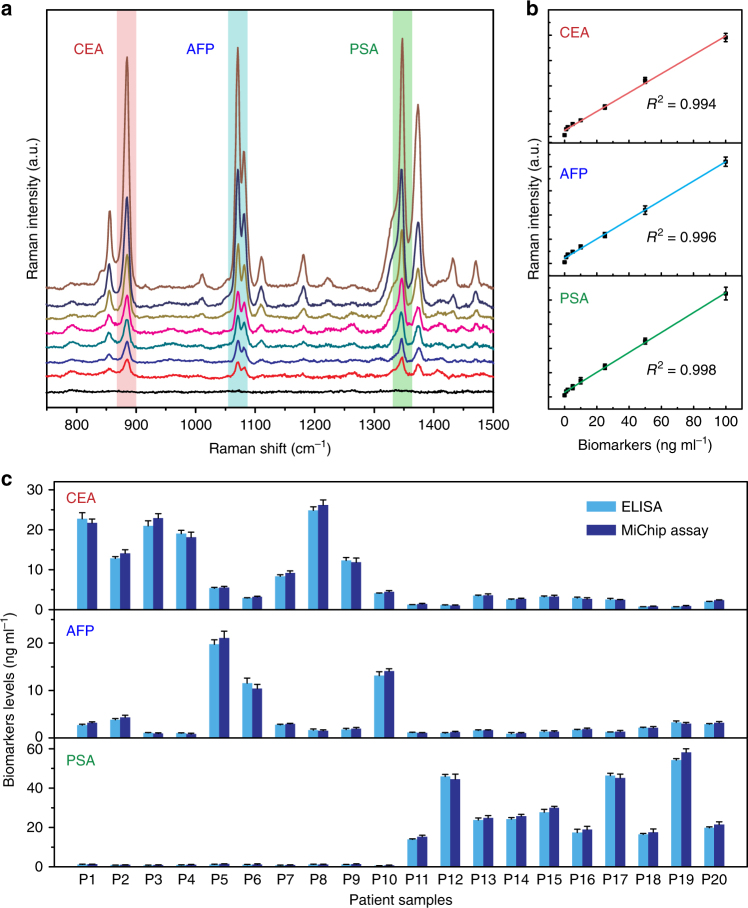
The MiChip assay of protein biomarkers in serum specimens. **a** SERS responses to a mixture biomarkers (PSA:AFP:CEA = 1:1:1, ranging from 0 to 100 ng ml^−1^) spiked in artificial serum samples. **b** Standard curves for the multiplexed quantitative analysis were generated by plotting the SERS peak intensity at 1341, 1071, and 885 cm^−1^ against the concentrations of PSA, AFP, and CEA, respectively. **c** The levels of the three biomarkers in the 20 clinical specimens measured by the MiChip assay and ELISA. The error bars in **b** and **c** stand for s.d. of triplicate measurements

We further examined the clinical utility of the MiChip assay by measuring the same panel of biomarkers in serum samples from 20 cancer patients diagnosed with prostate (*n* = 10), colorectal (*n* = 7) and hepatocellular (*n* = 3) cancers. The data of these clinical samples are summarized in Supplementary Table 1. The current biomarker calibration range (0.1–100 ng ml^−1^) evaluated by the MiChip assay is clinically relevant. For example, the commonly used serum cutoff values of PSA, AFP, and CEA are 4, 10, and 5 ng ml^−1^, respectively^36–38^. Simulated serum samples containing equal concentrations of PSA, AFP, and CEA in the range of 0–100 ng ml^−1^ were subjected for on-chip multiplexed analysis to establish calibration curves for each target. The SERS intensity at specific peaks retained its linear dependence on biomarker concentration (Fig. 4a, b) even under the multiplexed detection conditions. Importantly, the results showed that the MiChip assays were able to distinguish the three groups of cancer patients without any exception (Fig. 4c). For example, PSA concentrations varied from 15.3 to 58.1 ng ml^−1^ for the prostate cancer patients (P11-P20) with a mean of 30.2 ng ml^−1^. The PSA level of the colorectal and hepatocellular cancer patients fell into the range of 0.8 to 1.4 ng ml^−1^. PSA tests identified all the prostate cancer patients with good statistical accuracy (*P* < 0.0001). The results on detection of AFP (*P* < 0.0001) and CEA (*P* *=* 0.0003) are also consistent with the finding with PSA. We also validated the MiChip results using the standard ELISA commercial kit. The excellent linear correlation (*R*^2^ = 0.996) of the ELISA and MiChip results (Supplementary Fig. 21) confirm the ability of the MiChip platform to analyze complex clinical specimens.

### Rapid and multiplexed detection of bacteria

**Fig. 5:**
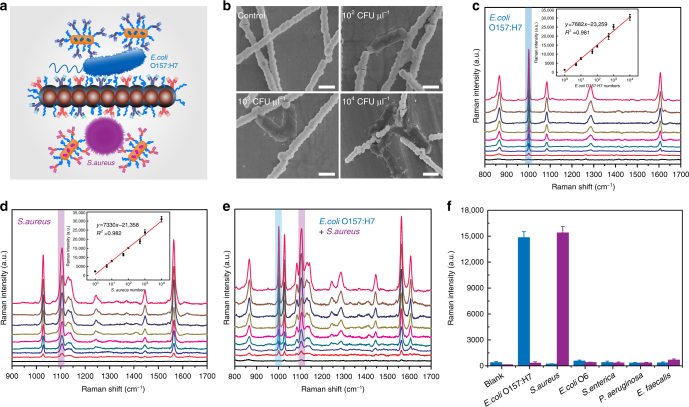
Multiplexed detection of bacteria by the MiChip assay. **a** Schematic illustration of the simultaneous detection of *E. coli* O157:H7 (blue) and *S. aureus* (purple). **b** SEM images of Magchains-*E. coli* O157:H7-SERS probes complex with varying concentrations of *E. coli* O157:H7. Scale bar: 1 μm. **c**, **d** SERS spectra of *E. coli* O157:H7 and *S. aureus* at different concentrations (ranging from 0 to 10^4^ CFU μl^−1^). Insets are calibration curves generated by plotting the SERS peak intensities at 1001.6 and 1104.9 cm^−1^ as a function of the logarithm of *E. coli* O157:H7 and *S. aureus*, respectively. **e** SERS responses to a mixture of the two pathogens (*E. coli* O157:H7: *S. aureus* = 1:1, ranging from 0 to 10^4^ CFU μl^−1^). **f** The MiChip assay results of saliva samples spiked with *E. coli* O157:H7, *S. aureus* and other control bacteria. The error bars in **c–f** represent the s.d. of triplicates (*n* = 3)

We next extended the MiChip assay to multiplexed identification and quantification of bacterial species (Fig. 5a). The rapid and sensitive detection of multiple infectious species such as bacteria is of considerable public interest for pinpointing the source of infection at the early stage and improving patient care and outcomes^5,39^. Magchains and SERS probes were prepared as described above. Here, the Raman reporter molecules, 3,5-difluorothiophenol (DFTP) and 2,4-dichlorothiophenol (DCTP) were chosen to prepare SERS probes specific to *Escherichia coli* O157:H7 (*E. coli* O157:H7) and *Staphylococcus aureus* (*S. aureus*) bacteria. Taking advantage of the Magchains for mixing and target separation allows us to detect bacteria on the same microchip that we used for protein biomarkers. We assayed saliva samples spiked with the bacteria. SEM images of samples collected from the detection chamber show that *E.coli* O157:H7 cells (Fig. 5b) and *S. aureus* (Supplementary Fig. 22) were conjugated with multiple Magchains and even more with SERS probes. For saliva samples containing *E. coli* O157:H7 (Fig. 5c) or *S. aureus* (Fig. 5d) or both (Fig. 5e), the SERS signals at the characteristic peaks (1001.6 cm^−1^ for *E. coli* O157:H7 and 1104.9 cm^−1^ for *S. aureus*) show consistent linear correlation with the bacterial concentration over four orders of magnitude ranging from 10^0^ to 10^4^ CFU µl^−1^ (insets in Fig. 5c, d). Given the capacity (1 µl) of the mixing chamber, this means that the MiChip assay offers a sensitivity that allows for detecting a single bacterium. Furthermore, screening among a panel of Gram-positive and Gram-negative bacteria (10^2^ CFU µl^−1^) led to strong signals from specific types of targets and negligible background from non-specific ones (Fig. 5f), demonstrating the high specificity for bacteria identification. The identification of infectious sources among a library of pathogenic species is critical for the selection of effective treatments. The MiChip assay can be easily customized into integrated kits for sensitively screening a panel of pathogens leading to similar symptoms within 8 min. In contrast, the slow turnaround of the current gold standard procedure such as bacterial culture remains a major challenge.

## Discussion

The complementary advantageous attributes of magnetic nanochains and SERS nanoprobes endow the MiChip with great versatility and flexibility for bioanalysis of liquid specimens. We have demonstrated the general utility and robustness of the MiChip platform by rapid, sensitive, accurate, and multiplexed cancer biomarker profiling in clinical serum samples and bacterial detection in human saliva, in contrast to the time-consuming procedures and low throughput of current clinical gold standards such as ELISA and bacterial culture^40,41^. The MiChip assay is highly sensitive because of two synergistic mechanisms: (1) the surface area of each AuNR is sufficient to accommodate more than 10^4^ Raman reporters for highly efficient signal amplification (EF > 10^5^); (2) magnetic separation from the mixing chamber (~2 × 10^5^ μm^2^) to the detection chamber (~2 × 10^3^ μm^2^) enriches the target by 100-fold. Rapid turnaround (8 min) of the MiChip assay enables timely response to incidents that need immediate medical attention, and also offers the technical basis for its use in detecting unstable targets and high-throughput screening^7,8,42^.

Liquid mixing has been a long-standing challenge in microfluidics because of the intrinsic laminar flow of spatially confined fluids^1,13^. In view of the widespread use of magnetic stirring in macroscopic systems, one would naturally consider it as a leading choice to promote liquid mixing in microfluidic devices. However, no magnetic mixing system has previously demonstrated effective uses in microfluidic functional assays. The polydopamine-based strategy produces robust nanochains with high loading of magnetic components, which leads to easy, reproducible manipulation of the nanochains by tailored magnetic fields for rotational and directional motion. This, together with the readily functionalized surface, allows the nanochains to serve as both tiny stir bars for active liquid mixing and capture agents for bioseparation. Compared with microfluidic platforms built upon passive chaotic mixers or active mixers driven by external stimuli^14,16^, the use of magnetic nanochains eliminates both the conventional mixers and target-specific biofunctionalized surfaces, making it possible to adopt a simple planar design of flat channels in the MiChip. As such, the MiChip free of any target-specific components becomes a generic consumable part for a wide spectrum of targets, with the magnetic control and Raman detection systems built off-chip for long-term uses. This design can effectively address common operational problems associated with complex channel designs such as clogging and poor reproducibility^43,44^, and also help reduce the assay cost.

The signal transduction based on SERS nanoprobes offers distinct advantages for multiplexed detection in unprocessed biofluids^24^. First, the sharp Raman peaks (full width at half maximum, FWHW = 1–2 nm) defined by molecular signatures makes it possible to develop a library of nanoprobes by encoding identical plasmonic substrates with molecular tags of different chemical structures^26^. These nanoprobes can be excited at a single wavelength and their SERS signals can be detected simultaneously for quantification of multiple targets^45^. The physical similarity of these probes makes it easier to modulate individual probes for comparable signal response against their targets, which is essential for easy quantification in multiplexed settings. Second, the plasmonic nanostructures dramatically amplify the signal of Raman reporters to allow for ultrasensitive single-particle detection. Raman enhancement by plasmonic substrates is most efficient for molecules in 1–2 nm of close proximity because the electromagnetic field surrounding the substrates rapidly decays beyond that range^46^. This effect and the intrinsically weak Raman scattering minimize the signal interference from external environment. Third, the scattering nature of the Raman signal imparts the nanoprobes with better photostability, compared with the commonly used fluorescent dyes^47^. Collectively, the SERS-encoded nanoprobes give rise to sensitive, stable, and spectrally resolved signals with low background noise. In the event of detecting a panel of biomarkers, fingerprint signals of the nanoprobes allow for simultaneous identification and quantification of multiple biomarkers in a single readout, unlike the spatial encoding used in most multiplexing systems.

The MiChip platform offers versatile opportunities to further extend its use for a broader range of targets and improve its sensitivity and multiplexing capacity. Taking into account the chemical flexibility to immobilize diverse types of proteins, nucleic acids, and small molecular affinity ligands on the Magchains and SERS probes, we anticipate applications of the MiChip assay for protein profiling and genotyping in a wide spectrum of subcellular (i.e., exosomes) and cellular targets^48^. Rapid, multiplexing signal readout of Raman spectroscopy is of considerable interest for real-time monitoring of interconnected targets, for example, the correlated protein markers in a signaling pathway. Recent advances in nanochemistry have opened the avenue to systematically optimize the spectral and structural details of plasmonic nanostructures for further improving the Raman enhancement efficiency and thus the sensitivity of the MiChip assay^49–52^. For example, the use of NTP-encoded Au@Ag core-shell SERS probe^53^ (Supplementary Fig. 23) that exhibits 40 folds of Raman intensity of the AuNR probe can further lower the detection of limit of PSA down to 0.2 pg ml^−1^ (Supplementary Fig. 24). Multiplexing capacity of the MiChip also can be increased by accommodating more distinguishable Raman reporters in the same spectral range. We have shown that 5 cancer biomarkers including PSA, AFP, CEA, CA125, and CA19-9 encoded by NTP, BTP, TFTP, DFTP, and DCTP can be quantitatively detected in a single spectral reading (Supplementary Fig. 25). Furthermore, the simple design of the current microfluidic system can be arranged in an array format to afford multiplicate capacity on a single chip, which further increases its throughput for translational applications, in conjugation with the micrometer spatial resolution of Raman spectroscopy. Also important is that both compact magnetic actuation systems and portable Raman spectroscopy are well-established to be coupled with microfluidic devices, enabling the development of automated, portable off-chip control/detection systems for on-site analysis.

## Methods

### Materials

All the standard proteins (PSA, CEA, AFP, CA125, and CA19-9) were purchased from Fitzgerald: PSA (30C-CP1017), CEA (30-1819), AFP (30-1029), CA125 (30-AC21), and CA19-9 (30-AC09). The following antibodies were used in this study (see Supplementary Table 2 for details): capture & detection antibody to PSA (GenScript), capture & detection antibody to CEA, AFP, CA125, and CA19-9 (Meridian, Life Science, Inc.), capture & detection antibody to *E. coli* O157:H7 (generated and provided by Dr. Weihua Lai), capture & detection antibody to *S. aureus* (Thermo Fisher). All bacteria were purchased from the American Type Culture Collection (ATCC). The bacterial strains used in the study were *E. coli* O157:H7 (ATCC 43888), *S. aureus* (ATCC 25923), *E. coli* O6 (ATCC 25922), *Salmonella enterica* (ATCC 13311), *Pseudomonas aeruginosa* (ATCC 142), and* Enterococcus faecalis* (ATCC 29212).

### Bacteria culture

Bacteria were seeded and cultured in suspension using the following media: *E. coli* O157:H7, *E. coli* O6, *S. enterica*, and *P. aeruginosa* were cultured in Luria–Bertani medium (LB, Oxoid, Basingstoke, UK) at 37 °C for 20 h; *S. aureus* and *E. faecalis* were cultured in Brain-Heart infusion broth (Oxoid, Basingstoke, England) at 37 °C. The concentration of *S. aureus* was determined by serial dilution with subsequent plating on agar plates and measurement of colony forming units (CFU). The cells were then treated with 0.3% formaldehyde for 24 h. The inactivated bacteria were collected by centrifugation at 4000 rpm and resuspended in 0.01 M PBS (pH 7.4). Finally, these bacteria were serially diluted to the desired concentrations with 0.01 M PBS (pH 7.4) for further use.

### Serum samples

The study was approved by the ethics committee of Tongji Medical College, Huazhong University of Science and Technology (IORG No. IORG0003571), and informed consent was obtained from all participants. Blood samples were collected from patients with prostate cancer (*n* = 10), hepatocellular carcinoma (*n* = 3) and colorectal cancer (*n* = 7). Serum was obtained via venipuncture into vacutainer tubes containing clot activator (BD Diagnostics, Franklin Lake, NJ). Cancer diagnoses were confirmed by traditional examination at Cancer Center, Union Hospital, Tongji Medical College.

### Synthesis of magnetic nanoparticles

The magnetic nanoparticles were synthesized by a modified solvothermal reaction^54^. Briefly, FeCl_3_ (0.54 g) and trisodium citrate (0.20 g) were first dissolved in ethylene glycol (20 ml), and sodium acetate (1.20 g) was added afterwards under stirring. The mixture was stirred vigorously for 30 min and then sealed in a Teflon-lined stainless-steel autoclave (30 ml capacity). The autoclave was heated at 200 °C and maintained for 10 h, and then allowed to cool to room temperature. The black products were washed five times with ethanol and deionized (DI) H_2_O.

### Preparation of antibody-conjugated Magchains

To prepare the magnetic nanochains, 1 mg magnetic nanoparticles were dispersed in 18 mL bicine buffer (pH 8.5, 10 mM), followed by adding 8 mg dopamine. The mixture was immediately placed in a uniform magnetic field (0.05 T) for 15 min and then left undisturbed for 12 h. After the incubation, the product was separated with a permanent magnet and dispersed in 1 ml of DI H_2_O. The length and width of the resulting magnetic nanochains were controlled by changing the magnetic reaction time and the initial dopamine concentration (Supplementary Fig. 12). The average length of the nanochains grew from 10 to 20 μm when the reaction time in the magnetic field was extended from 10 to 30 min. The nanochains formed at this stage were fragile and easily broke into shorter fragments of ~3 μm after 3 s of ultrasonication, allowing for preparation of shorter nanochains. Subsequent reaction in absence of the magnetic field led to permanently fixed nanochains by continuous deposition of polydopamine. The width of the nanochains is primarily dependent on the initial concentration of dopamine, with concentrations of 0.2, 0.45, and 0.9 mg ml^−1^ leading to nanochain widths of 300, 400, and 600 nm, respectively.

The harvested nanochains were washed three times with DI H_2_O and dispersed in 5 ml bicine buffer (pH 8.5, 10 mM). 100 μl antibody solution (Ab-I, 0.25 mg ml^−1^) was added to the dispersion and the mixture was shaken for 12 h at room temperature. Afterwards, 1 ml mPEG-SH solution (10 mg ml^−1^) was added to the mixture to block the unreacted sites for 12 h. The excess reactants were removed by repeated magnetic separation and washing. Finally, the bioconjugated magnetic nanochains were resuspended in 1 ml PBS (0.01 M, pH 7.4) and stored at 4 °C before use.

### Synthesis of AuNRs

A seed-mediated method was used to prepare the AuNRs^55^. First, 0.60 ml of ice-cold NaBH_4_ (10 mM) was quickly added to 10 ml of HAuCl_4_ (0.25 mM) dissolved in 0.2 M CTAB surfactant solution. The mixture was stirred for 2 min, producing a brownish yellow solution of small gold seeds. The seed solution was kept for at least 1 h at 30 °C before it was used in the next step. Second, AuNRs were synthesized in a growth solution. 40 μl of AgNO_3_ (0.1 M) was added to 10 ml of HAuCl_4_ solution (1.25 mM) in 0.1 M CTAB. 0.5 ml of hydroquinone aqueous solution (0.1 M) was then introduced to reduce HAuCl_4_ to colorless HAuCl_2_, followed by the injection of 0.16 ml of seed solution to initiate the growth of AuNRs. The growth solution was mixed thoroughly for 2 min and kept undisturbed in a water bath at 30 °C for 12 h. The AuNRs were purified three times by centrifugation (5000 rpm, 30 min) and were dispersed in 10 ml of DI H_2_O.

### Preparation of SERS probes

The AuNRs were encoded with different Raman reporter molecules using the following procedure. First, 2 ml of AuNRs solution was centrifuged at 5000 rpm for 10 min and redispersed in 2 ml of dimethyl formamide (DMF). Then, 200 μl of Raman reporter molecules (20 mM in DMF) were added to the solution and the reaction mixture was stirred for 2 h. In this process, Raman molecules anchored on the surface of AuNRs via the Au-S bond. The SERS-encoded AuNRs were then harvested by centrifugation at 5000 rpm for 10 min and redispersed in 2 ml of fresh DMF. A mixture of mPEG-SH (50 μl, 1 mM, MW = 5000) and HOOC-PEG-SH (300 μl, 1 mM, MW = 3400) was added to the mixture and reacted for 1 h, resulting in a mixed layer of mPEG-SH and HOOC-PEG-SH on the AuNRs surface. Finally, the PEGylated AuNRs were centrifuged twice at 5000 rpm for 10 min and resuspended in 2 ml of DI H_2_O.

The harvested SERS-encoded AuNRs were conjugated with different antibodies using a similar protocol. Briefly, the carboxyl functionalized AuNRs were collected by centrifugation and dispersed in 4 ml of MES buffer (0.01 M, pH 5.5). Next, 0.2 ml of 1-ethyl-3-(3-dimethylaminopropyl) carbodiimide (EDC, 5 mg ml^−1^) and sulfo-*N*-hydroxysuccinimide (sulfo-NHS, 5 mg ml^−1^) were quickly added and incubated for 30 min to activate the carboxylic acid groups. The excess EDC and sulfo-NHS was removed by centrifugation, and 20 μg of detection antibody (Ab-II) in 2 ml of borate saline buffer (0.01 M, pH 8.5) was introduced immediately to the activated particles and the mixture was reacted under gentle stirring for 3 h at room temperature. Finally, 0.2 ml of bovine serum albumin (BSA) solution (10 mg ml^−1^) was introduced to the mixture for surface blocking. After 1 h, the SERS probes were centrifuged to separate the free reactants. The PEGylated SERS nanoprobes show good stability even in high salt and protein solutions (Supplementary Fig. 10).

### Fabrication of microfluidic chip

The microfluidic system was fabricated by stacking two patterned PDMS layers on a glass slide^56^. The bottom PDMS layer contains the fluid channels while the top layer hosts the pneumatic control channels. Protruding mold patterns for these channels (50 µm thickness) were fabricated via conventional photolithography on silicon wafers with SU-8 50 photoresist (Microchem). PDMS prepolymer and curing agent (Sylgard 184, Dow Corning) were mixed in ratios of 20:1 and 5:1 (w/w) and degassed in a vacuum chamber. The 20:1 PDMS mixture was spun on the fluid channel mold at 1500 rpm for 60 s while the 5:1 PDMS mixture was poured on the control valve mold. Both molds were then baked at 80 °C for 30 min. The PDMS layer (3 mm in thickness) that hosts the pneumatic control valves was peeled off from its mold. Access holes were made for pressure control with a hole puncher and the control valve layer was aligned to the fluid channel layer using a customized alignment tool. The two layers were bonded together by baking the assembly at 80 °C for 2 h, after which the structure was peeled off from the fluid channel mold. After punching the inlet/outlet holes for fluid access, the chip was bonded to a glass slide through the oxygen plasma treatment.

### Electromagnetic coils setup

The working principle of the electromagnets providing a rotating magnetic field is similar to a two phase brushless DC motor. The four coils are mounted on a cross-shaped aluminum plate. Each coil consists of windings maximizing the space allowed by a quadrant on the plate. Ferrite cores were inserted in the coils to increase the magnetic flux. A function generator supplies the reference pulse trains for a stepper motor driver to generate the two phases of high current excitation at a microstepping setting of 1/128 to approximate a sinusoidal waveform. The coils generate a rotating magnetic field with adjustable frequencies in the range of 1–11 Hz (60–660 revolutions per minute). No cooling measure was taken because of the minimal temperature increase (<1.5 ^o^C) at the microchip during the operation of 1–5 min (Supplementary Fig. 26).

### Evaluation of target capturing efficiency

Overall, 0.25 mg antibody-conjugated Magchains were mixed with 1 ml of relevant biomarker spiked sample on a stir plate for 1 min. Subsequently, the mixture was incubated without disturbance for 15 min and separated by a magnet. The capture efficiency was calculated as the ratio of the targets captured by the nanochains to the total amount of targets originally present. For the measurement of molecular biomarkers (PSA, CEA, and AFP), the amount of captured molecules was determined using commercial ELISA kits. For the bacterial (*E. coli* O157:H7 and *S. aureus*) measurement, the amount of captured bacteria was determined by plate counting.

### Detection of biomarkers on the MiChip

For the detection of cancer protein biomarkers, 1 μl of standard samples (0.1–100 ng ml^−1^) or clinical serum samples and 1 μl of detection reagents (Magchains and SERS probes) were injected into the MiChip platform. The two streams were then mixed within the mixing chamber, and followed by target recognition, separation, and Raman measurements. Similarly, bacteria of a range of concentrations (10^0^ to 10^4^ CFU μl^−1^) and bacteria spiked saliva were tested using the MiChip platform. All measurements were done in triplicate, and data are shown as mean ± s.d. An illustration of the entire setup is presented in Supplementary Fig. 27.

### Enzyme-linked immunosorbant assay (ELISA)

The concentration of cancer biomarkers (PSA, CEA and AFP) was measured using commercial ELISA kits: PSA (IB19126, IBL-America), AFP (IB19102, IBL-America), and CEA (RAB0411, Sigma-Aldrich). The assays were conducted according to instructions for use. Briefly, 25 μl of standards, controls and unknown samples were introduced to selected wells. Afterwards, 100 μl of conjugate reagent was added to all wells and incubated for 30 min at room temperature. The wells were subsequently washed three times with 0.01% Tween-20 in PBS (PBST, pH = 7.4), and horseradish peroxidase (HRP)-conjugated secondary antibody was added to all wells and incubated for 30 min. The reaction wells were then washed five times with PBST followed by 15 min incubation in 100 μl of 3,3′,5,5′-tetramethylbenzidine reagent. Finally, all wells were supplemented with 50 μl of stop solution (2 M H_2_SO_4_) and analyzed for absorbance at 450 nm.

### Characterization analysis

Scanning electron microscopy (SEM) images were captured using a field-emission scanning electron microscope (JSM-6700F). Transmission electron microscopy (TEM) observations were conducted on a Jeol JEM 2010 electron microscope at an acceleration voltage of 300 kV. SERS experiments were conducted using a Renishaw Raman microscope equipped with 633/785 nm excitation lasers. The laser beam with a laser spot size of 2–5 μm was focused by a ×50 objective. A single scan with an integration time of 15 s was performed for each spectrum acquisition.

### Data availability

The authors declare that the data supporting the findings of this study are available within the article and its supplementary information or from the corresponding author upon reasonable request.

## Electronic supplementary material

Supplementary Information

Description of Additional Supplementary Files

Supplementary Movie 1

Supplementary Movie 2

Supplementary Movie 3

Supplementary Movie 4
