# Supplementary material for: Magnetic nanochain integrated microfluidic biochips
Source: Nat Commun. 2018 May 1;9:1743. doi: 10.1038/s41467-018-04172-1 (PMC5931612; doi:10.1038/s41467-018-04172-1)
Supplement: Supplementary file 1 — Supplementary Information [file 41467_2018_4172_MOESM1_ESM.pdf]

# Supplementary Information

# Magnetic nanochain integrated microfluidic biochips

Xiong *et al*.

**Supplementary Figures**

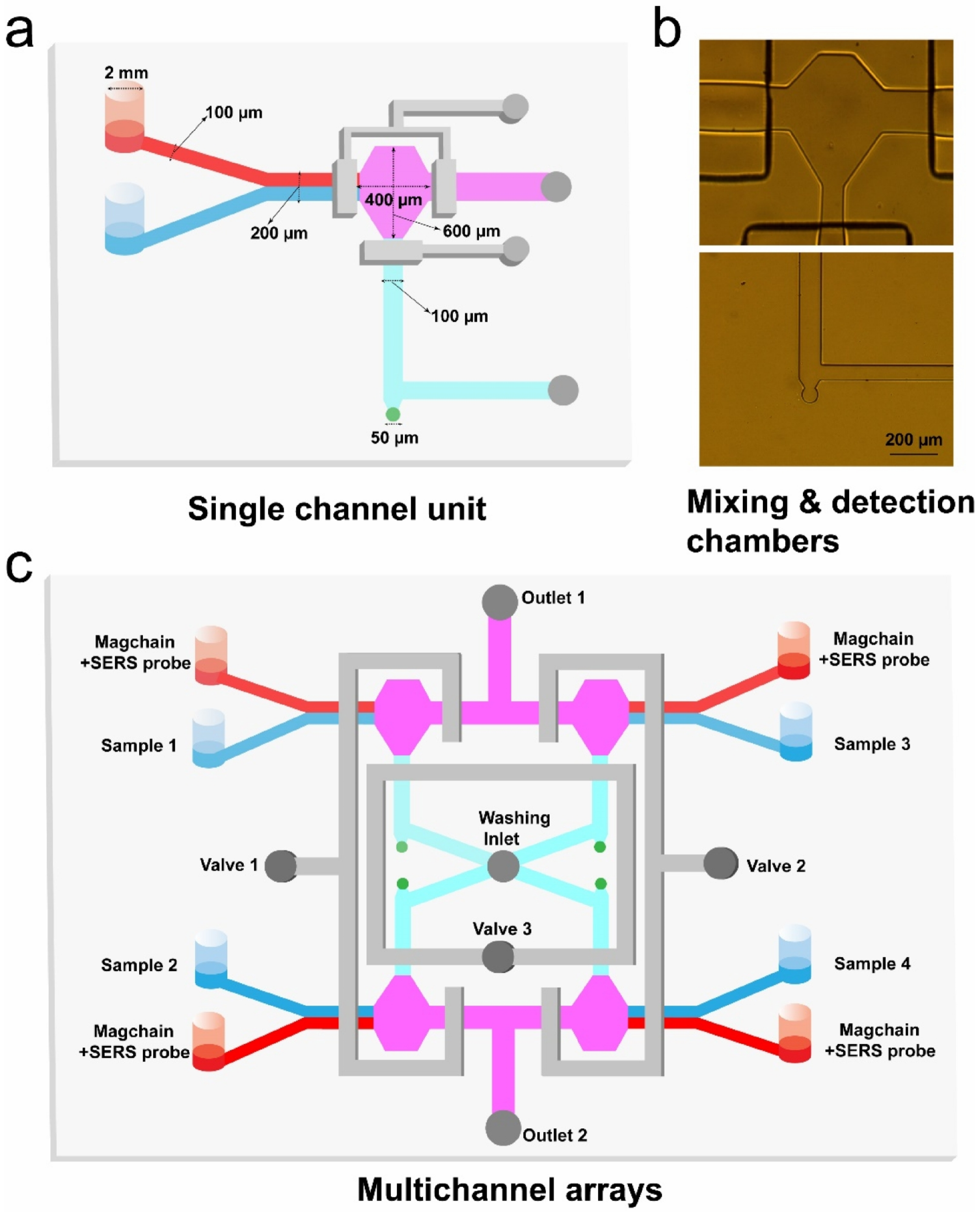


**Supplementary Figure 1. a,** Design of the single assay microfluidic chip. The dimensions of channels and chambers are labeled. **b**, The bright field images of mixing chamber and detection chamber. **c**, Schematic of a multichannel array for parallel analysis of four samples simultaneously.

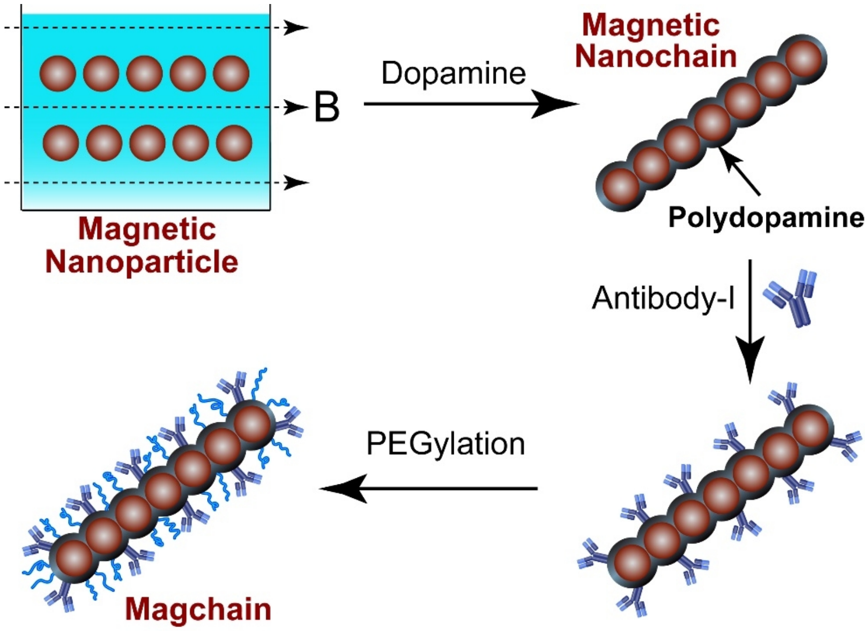


**Supplementary Figure 2.** Schematic illustration of the stepwise preparation of functionalized magnetic nanochains via magnetic alignment of magnetic nanoparticles, self-polymerization of dopamine to crosslink the nanochains, and subsequent antibody conjugation and PEGylation on the surface of nanochains.

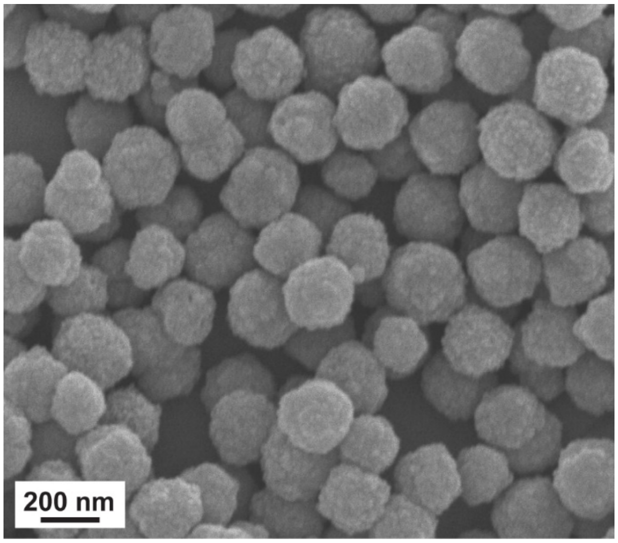


**Supplementary Figure 3.** SEM image of magnetic nanoparticles with an average diameter of 250 nm.

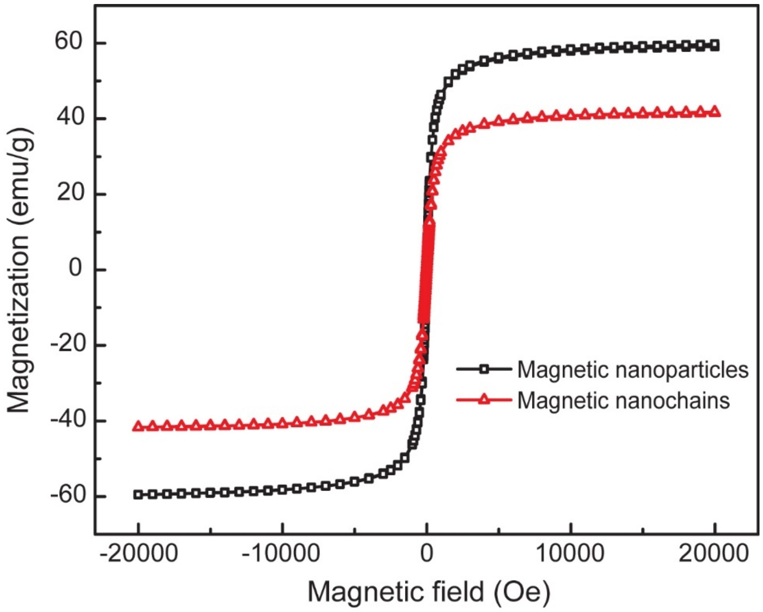


**Supplementary Figure 4.** Hysteresis loops of as-synthesized magnetic nanoparticles and magnetic nanochains. Magnetization saturation values (Ms) of the magnetic nanoparticles and magnetic chains are 59.7 and 41.7 emu $g^{-1}$, respectively.

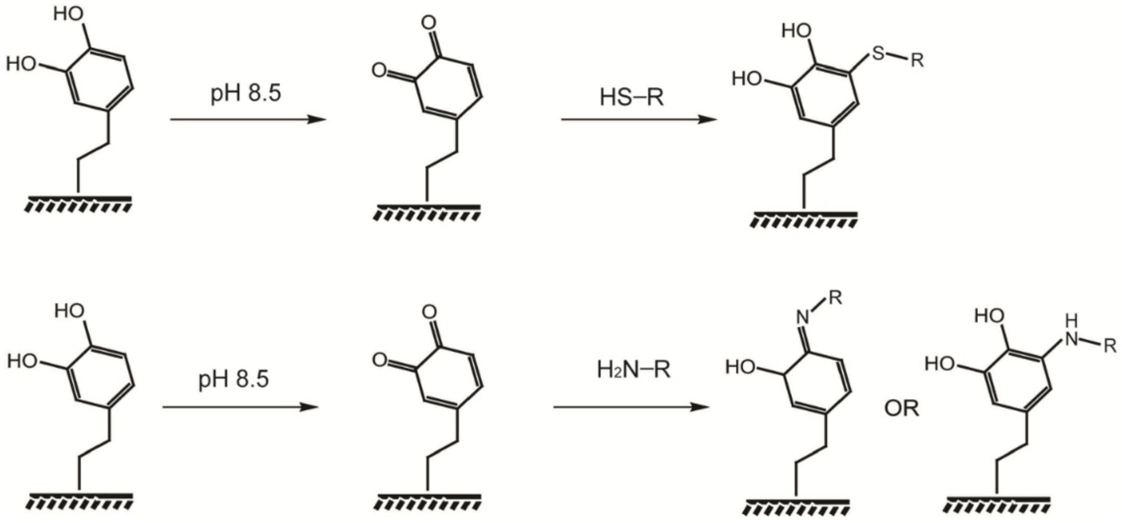


**Supplementary Figure 5.** The reactions of polydopamine with thiol and amine groups *via* Michael addition and/or Schiff base reactions.

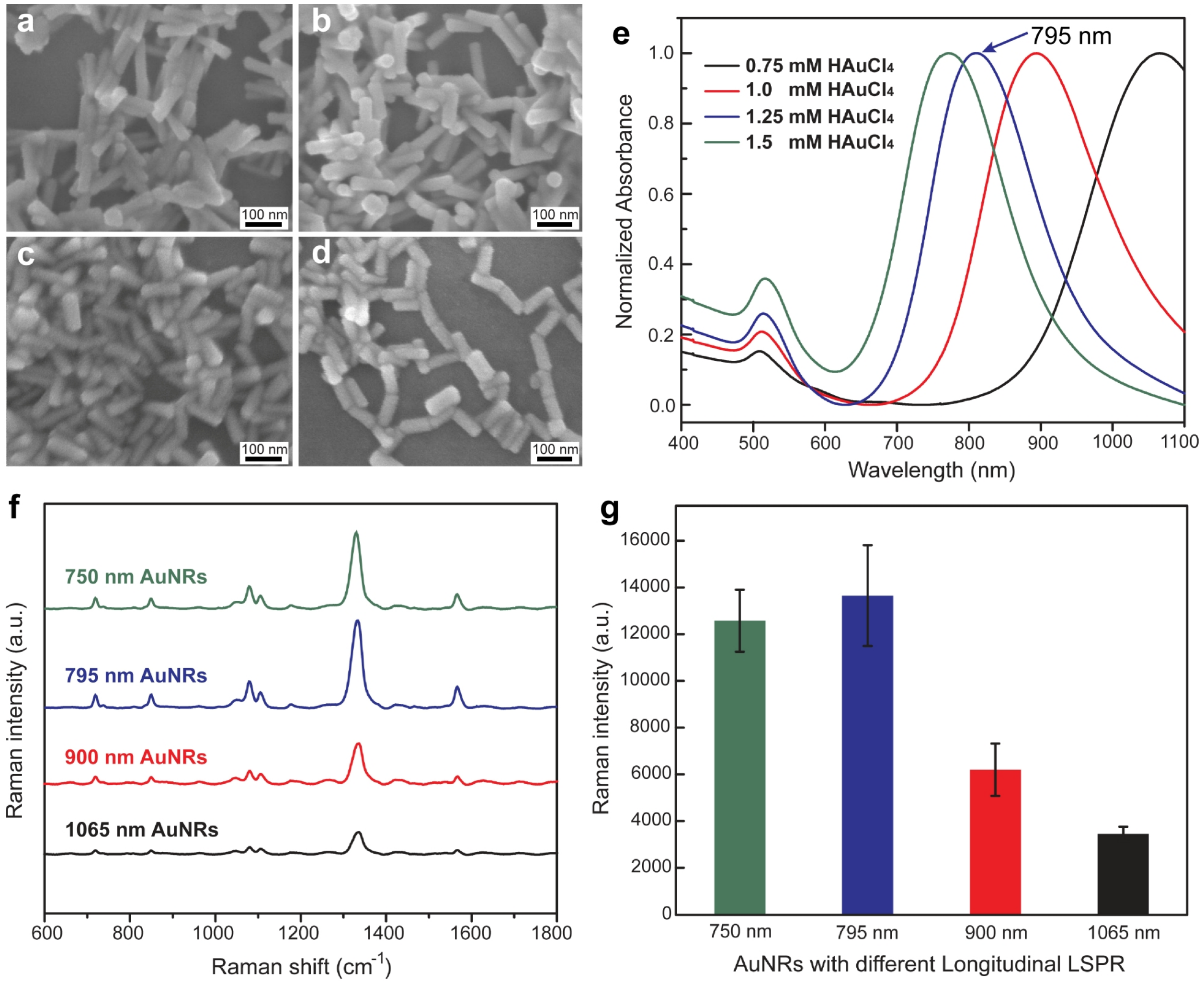


**Supplementary Figure 6.** The preparation of AuNRs. **a - d**, SEM images of AuNRs synthesized at different concentration of $HAuCl_4$: 0.75 mM (**a**), 1.0 mM (**b**), 1.25 mM (**c**), 1.5 mM (**d**). **e**, Longitudinal localized surface plasmon resonance (LSPR) of AuNRs. Au NRs with a range of aspect ratios from 3.5 to 6.8 and LSPR from 750 to 1065 nm were obtained by changing $HAuCl_4$ concentration. AuNRs with a longitudinal LSPR at 795 nm, which is close to the laser excitation wavelength of 785 nm, were chosen for SERS probe preparation. **f**, SERS spectra of 4 representative 4-nitrothiolphenol (NTP)-encoded AuNRs with different longitudinal LSPR. **g**, SERS intensity at 1341 $cm^{-1}$ of the NTP-encoded AuNRs with different LSPR peaks.

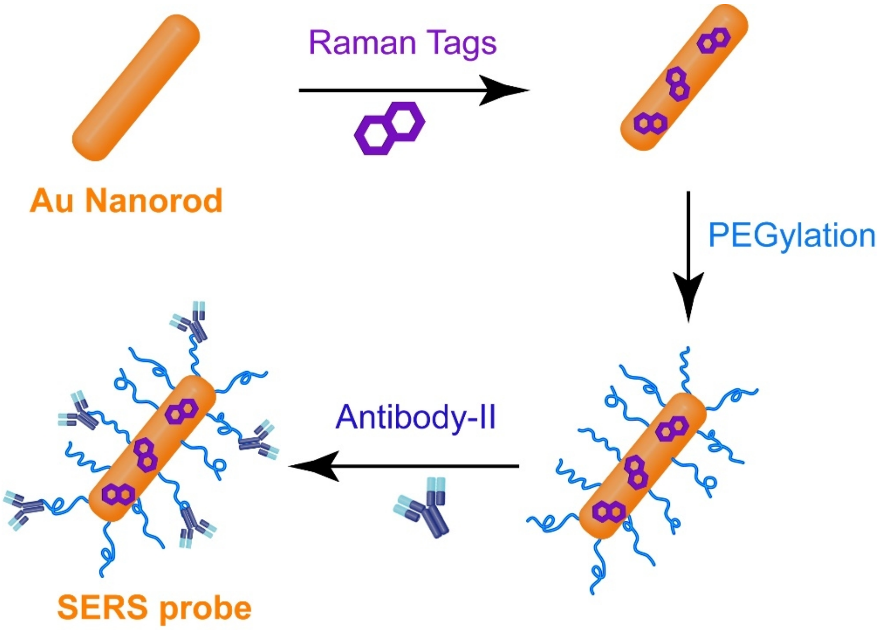


**Supplementary Figure 7.** Schematic illustration of the preparation of SERS-encoded nanoprobes in stepwise anchoring of Raman reporter molecules on the AuNRs, PEGylation, and antibody conjugation.

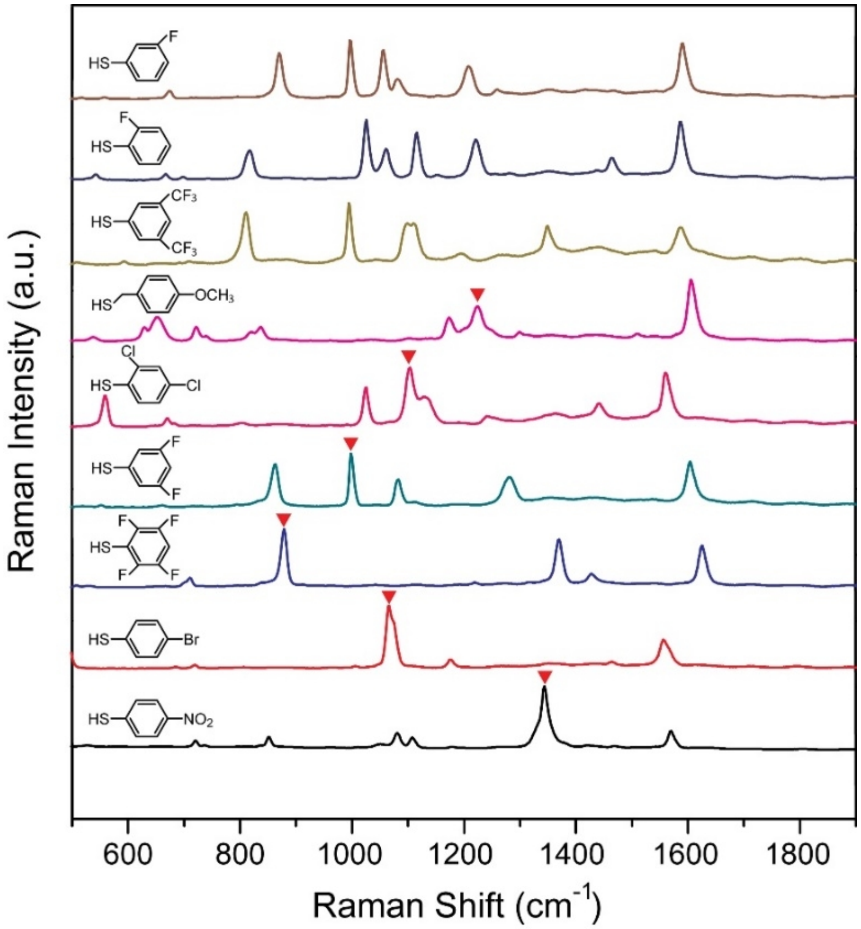


**Supplementary Figure 8.** SERS spectra of 9 representative SERS-encoded AuNRs. From bottom to top: 4-nitrothiophenol (NTP), 4-bromothiophenol (BTP), 2,3,5,6-tetrafluorothiophenol (TFTP), 3,5-difluorothiophenol (DFTP), 2,4-dichlorothiophenol (DCTP), and 4-methoxy-α-toluenethiol (MATT). 3,5-bis(trifluoromethyl)benzenethiol (FMBT), 2-fluorothiophenol (2-FTP), 3-fluorothiophenol (3-FTP). The well-resolved peaks used for multiplexed detection are labeled.

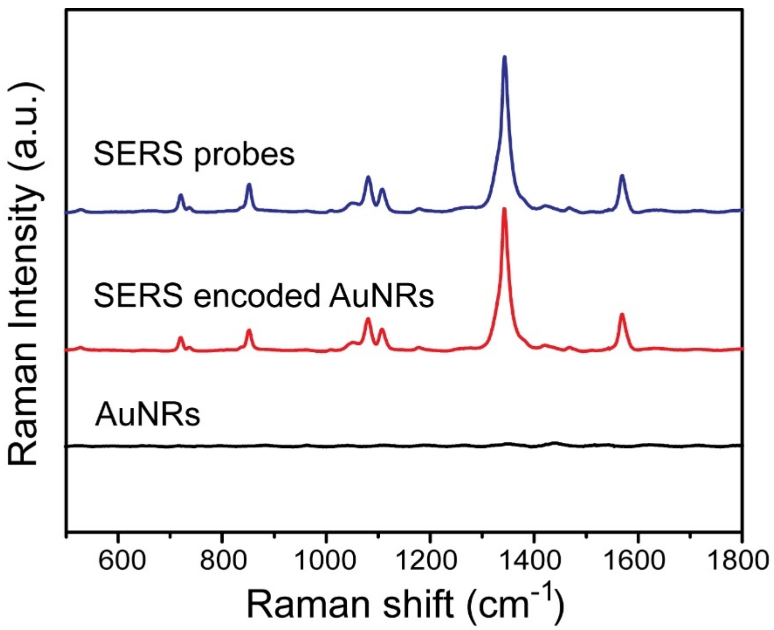


**Supplementary Figure 9.** Characterization of SERS probe. From bottom to top: Raman spectra of naked AuNRs, SERS-encoded AuNRs and antibody-conjugated SERS probes. The probe exhibits strong SERS signal after Raman molecules tagged on the AuNRs and the SERS intensity is stable after the antibody conjugation.

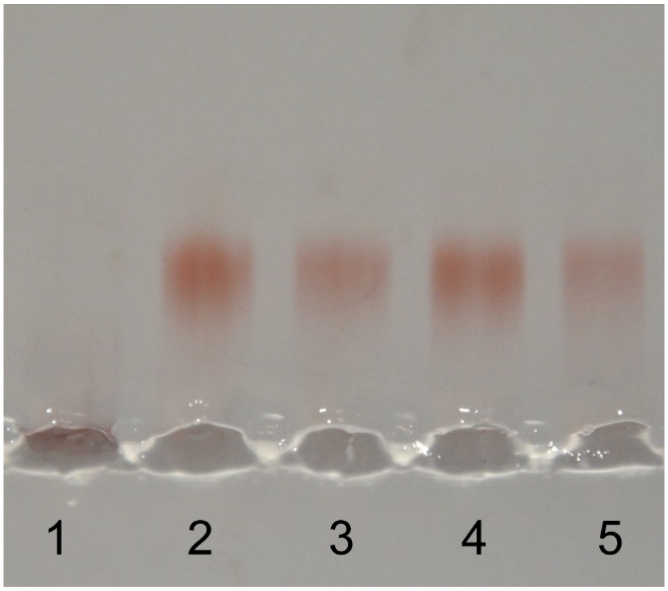


**Supplementaray Figure 10.** Agarose gel electrophoresis analysis of the stability of SERS probes. **Lane 1,** original AuNRs. **Lane 2**, PEGylated SERS probes. **Lane 3-5**, PEGylated SERS probes that incubated with 0.01 M of PBS (**lane 3**), 5% of NaCl (**lane 4**), and 10% of BSA (**lane 5**) for 0.5 h. The results show the naked AuNRs were aggregated in the electrophoretic buffer solution and the PEGylated SERS probes are stable even in high salt and protein solutions.

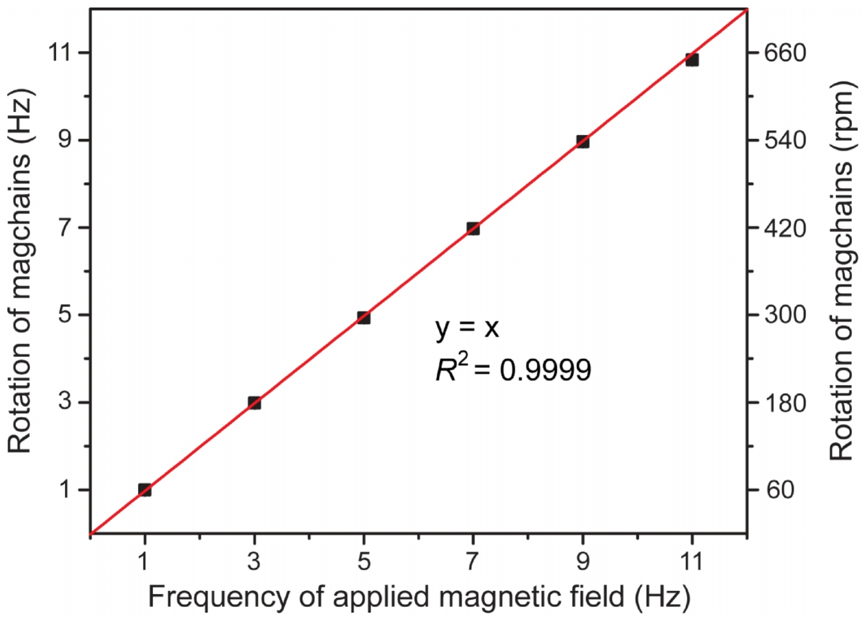


**Supplementary Figure 11.** Rotation frequency of magnetic nanochains observed by time-lapse high-speed camera. The rotating magnetic nanochains (20 µm in length and 400 nm in width) in the alternating magnetic field (Frequency ranged from 1-11 Hz) were imaged by a high speed camera (500 frames per second). The rotation frequency of magnetic nanochains was calculated from the captured images in 5 s. The results show the magnetic chain can maintain synchronous rotation up to 9 Hz (540 rpm). The magnetic chain starts to exhibit a small degree of hysteresis at an applied magnetic field of 11 Hz.

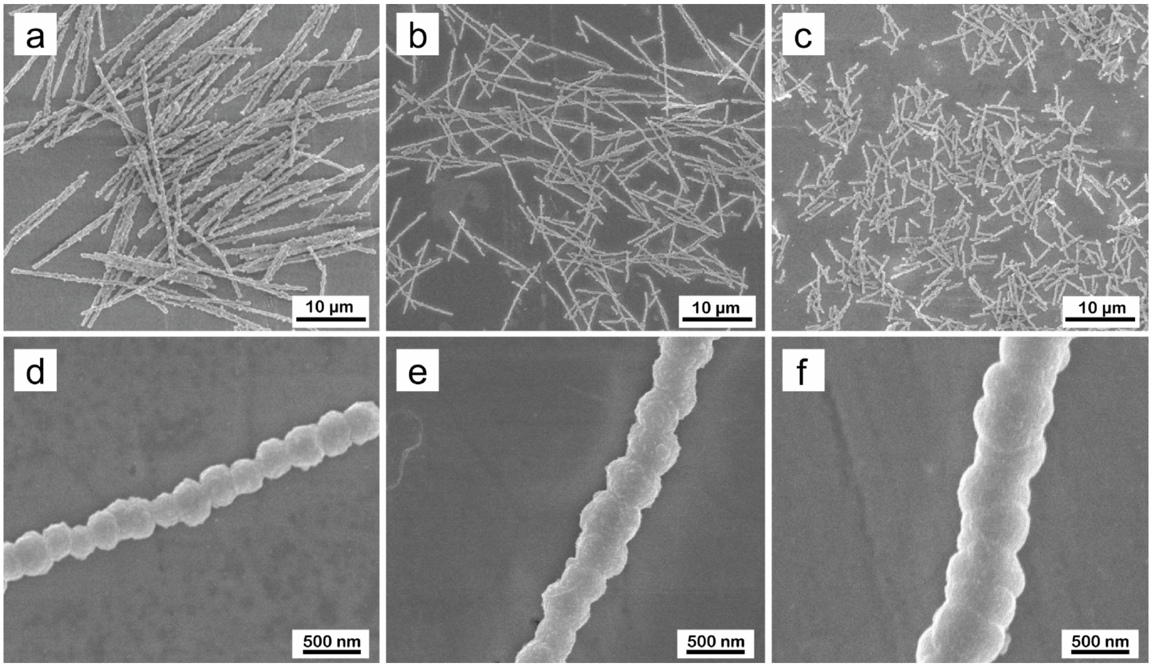


**Supplementary Figure 12.** SEM images of magnetic nanochains of different length and width. **a - c**, 20, 10, and 3 μm in length, respectively. **d - f**, 300, 400, and 600 nm in width, respectively.

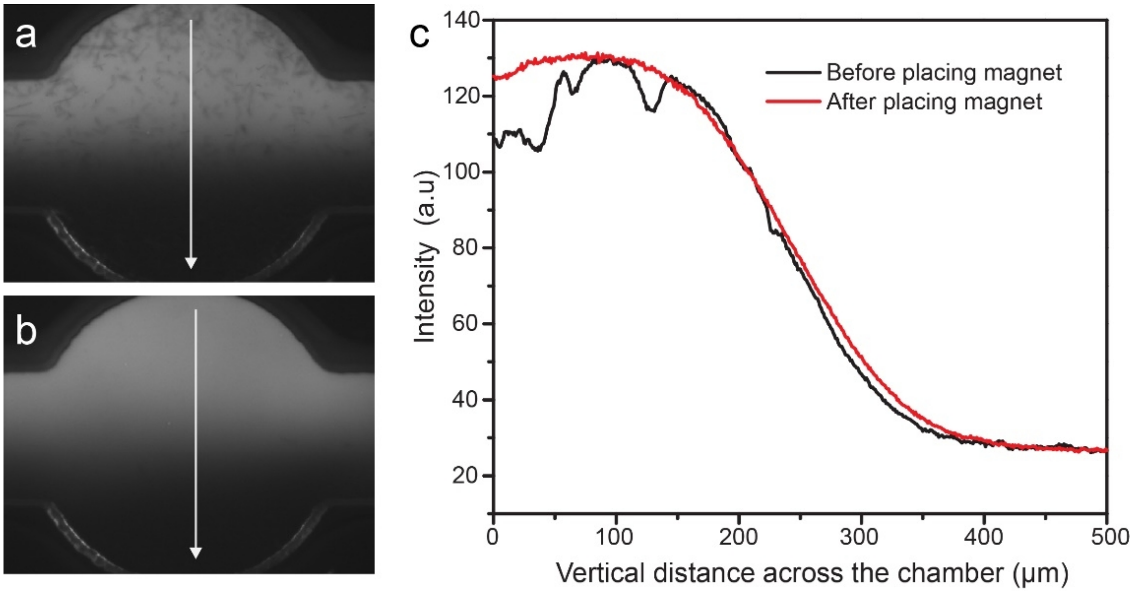


**Supplementary Figure 13.** Elimination of the impact of magnetic chain images in the evaluation of mixing efficiency. **a**, Image of mixing chamber before placing a magnet. **b**, Image of mixing chamber after placing a magnet. **c**, Fluorescent intensity profiles evaluated across the centre of the mixing chamber.

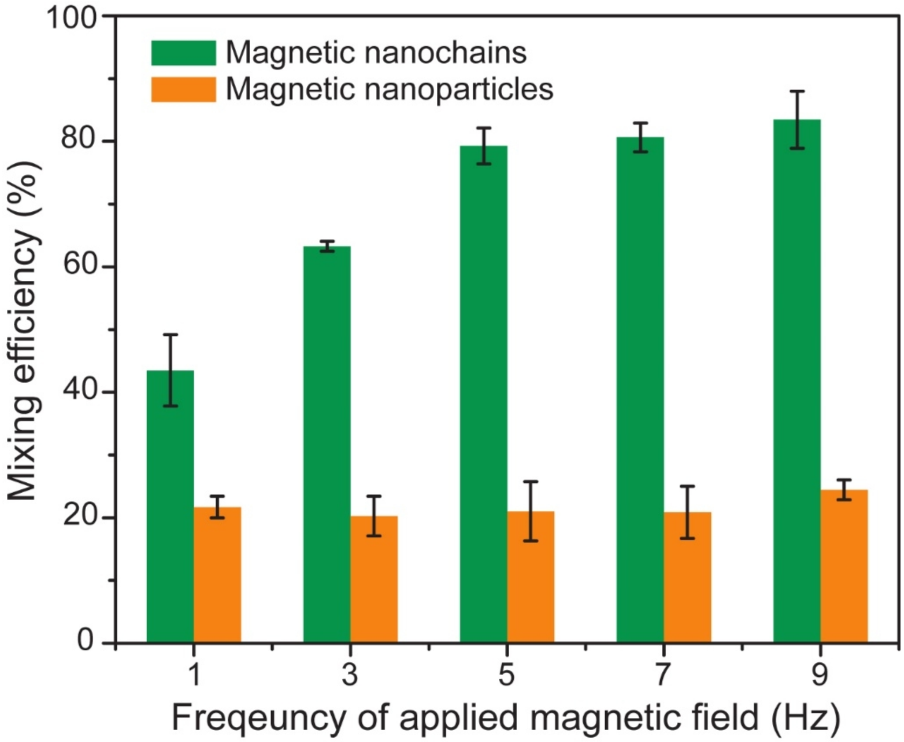


**Supplementary Figure 14.** Mixing efficiency of magnetic nanochains and magnetic nanoparticles in spinning magnetic fields of different frequency. The mixing efficiency of magnetic nanochains and magnetic nanoparticles at different magnetic fields was evaluated in 1 min. The results show the mixing efficiency of magnetic nanochains increases with increasing spinning frequency and level off at 5 Hz (300 rpm). In contrast, magnetic nanoparticles of the same concentration only led to 21% of mixing, which is similar to the control sample in absence of the alternating magnetic field.

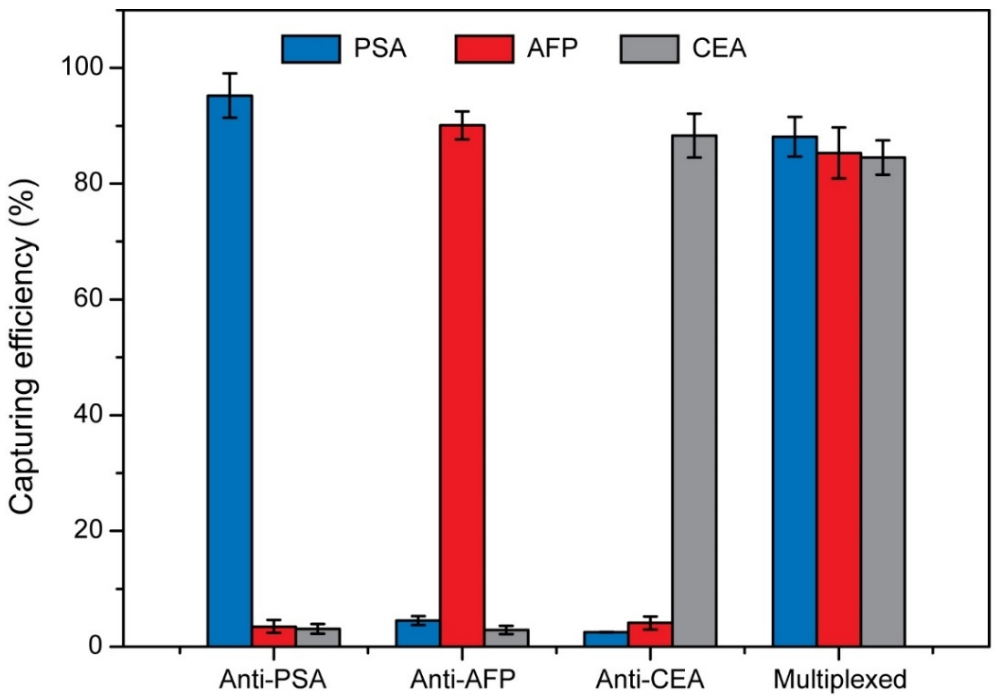


**Supplementary Figure 15.** Capture efficiency of cancer biomarkers by magnetic nanochains. A mixture of cancer biomarkers (PSA, CEA and AFP) were incubated with magnetic nanochains (functionalized with either anti-PSA, anti-CEA, anti-AFP or a mixture of the three antibodies). The achieved capture efficiency was determined using ELISA upon magnetic separation. The magnetic chains show high capture efficiency (90-95%) against the relevant targets with low crosstalk against the mismatched targets (< 5%). The multiplexed nanochains can simultaneously capture the three targets with a slight drop of capture efficiency (85-88%).

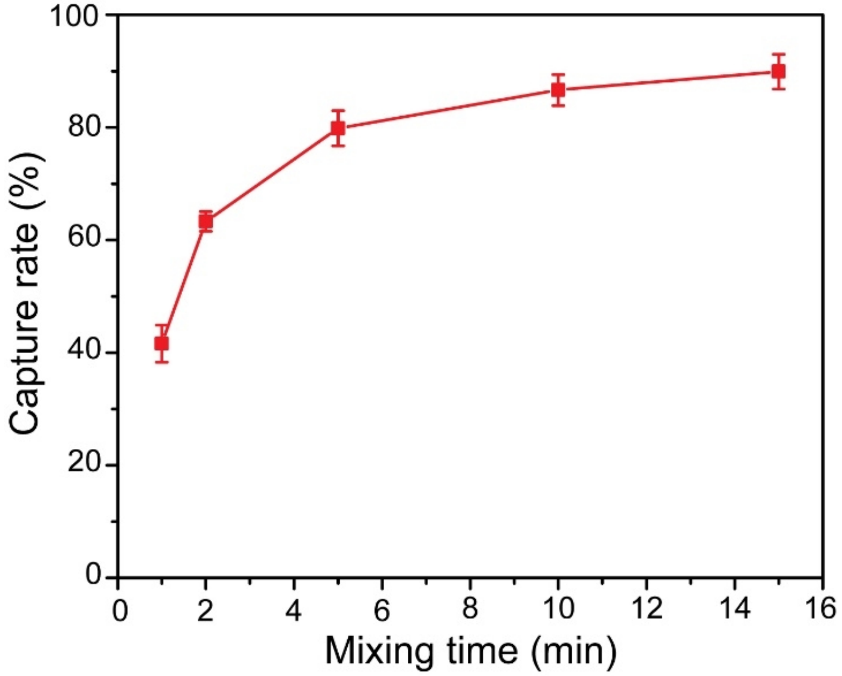


**Supplementary Figure 16.** Capture rate of PSA versus mixing time. PSA (100 ng $ml^{-1}$) was mixed and reacted with anti-PSA magnetic nanochains. The capture rates were determined by ELISA.

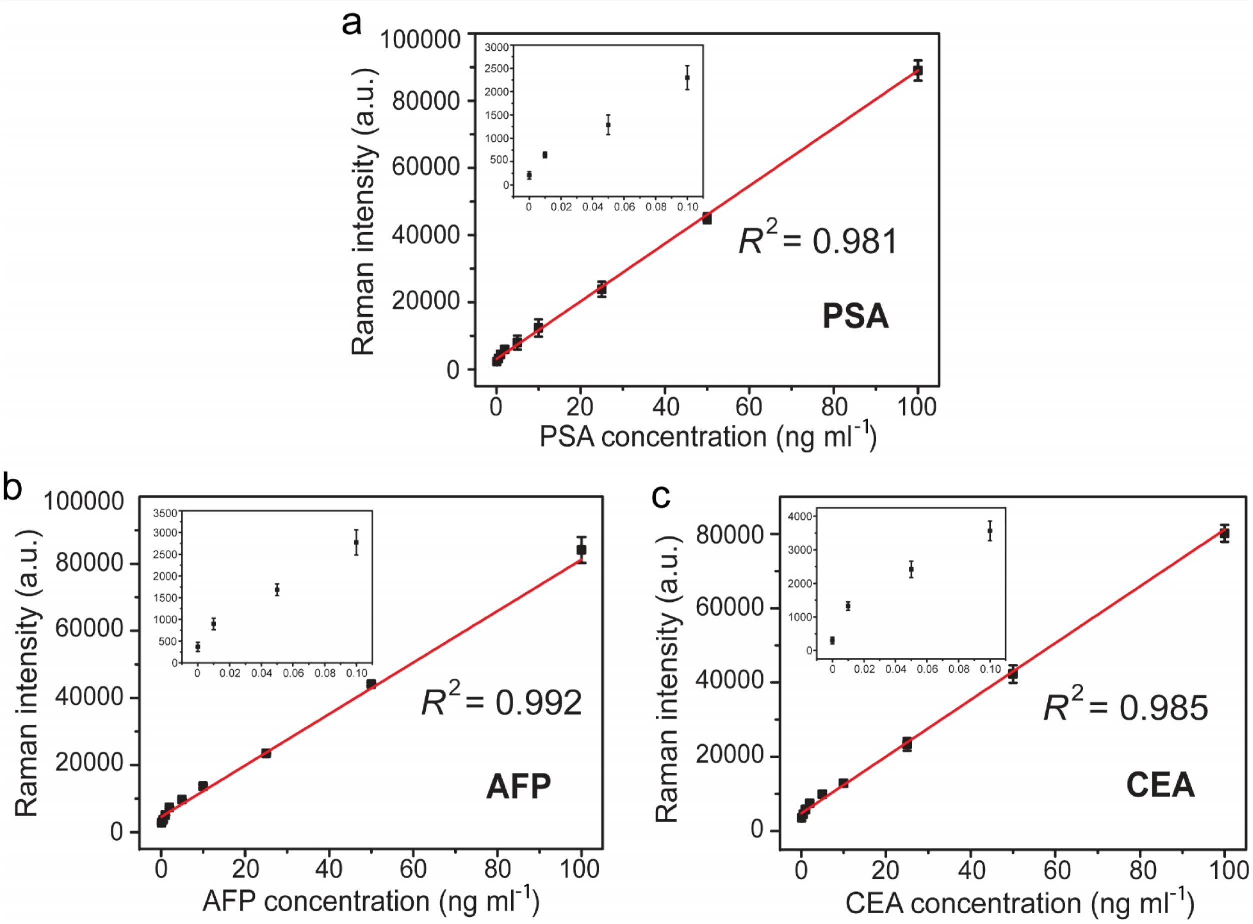


**Supplementary Figure 17.** Dose-response calibration curves for PSA (**a**), AFP (**b**) and CEA (**c**) in PBS buffer solution based on the MiChip assay. The SERS intensity of signature peaks as a function of the concentration of PSA (at 1346.5 $cm^{-1}$), AFP (at 1070.7 $cm^{-1}$) and CEA (at 884.9 $cm^{-1}$). Insets: calibrations curves at low concentrations (PSA, AFP and CEA ranged from 0 to 0.1 ng $ml^{-1}$). The SERS assay shows a good linearity ($R^2$ > 0.98) across a wide concentration range. The LOD is estimated to be 10 pg $ml^{-1}$ for PSA, AFP and CEA assessed from 3 times s.d. of blank signal. The error bar stands for s.d. of three replicates.

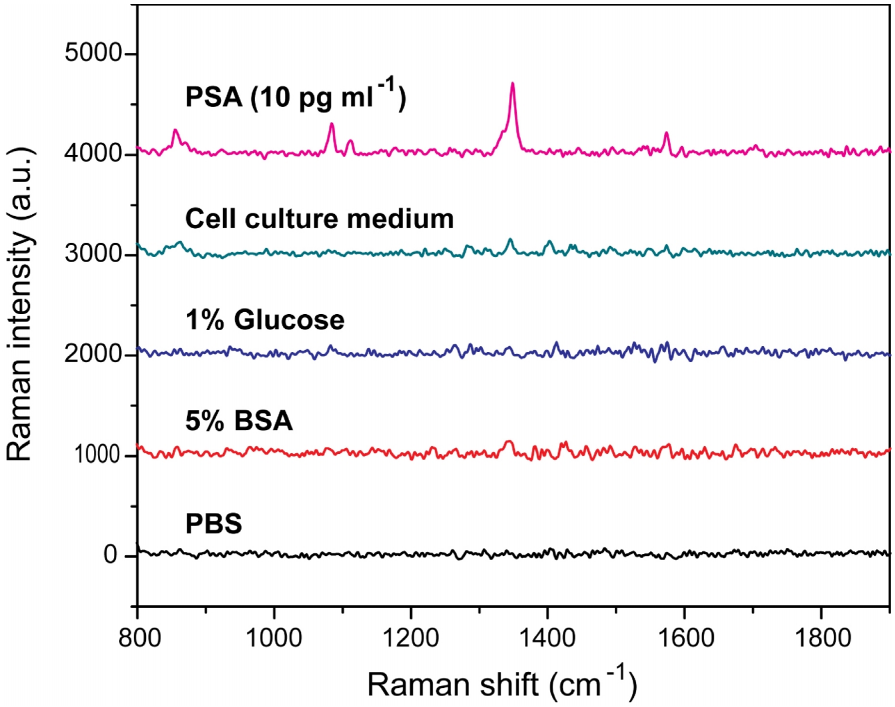


**Supplementary Figure 18.** Specificity of the MiChip assay. The assay shows minimal non-specific binding/background signal caused by PBS, 5% BSA, 1% glucose and cell culture medium (10% FBS).

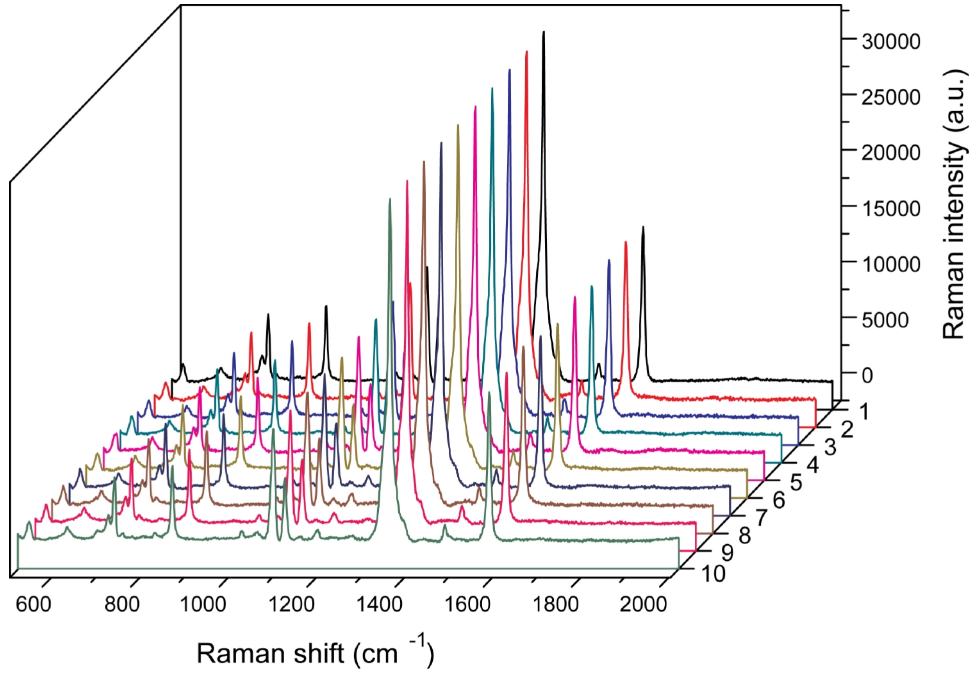


**Supplementary Figure 19.** SERS spectra of NTP-encoded AuNR SERS probe acquired in 10 continuous measurements at the same spot.

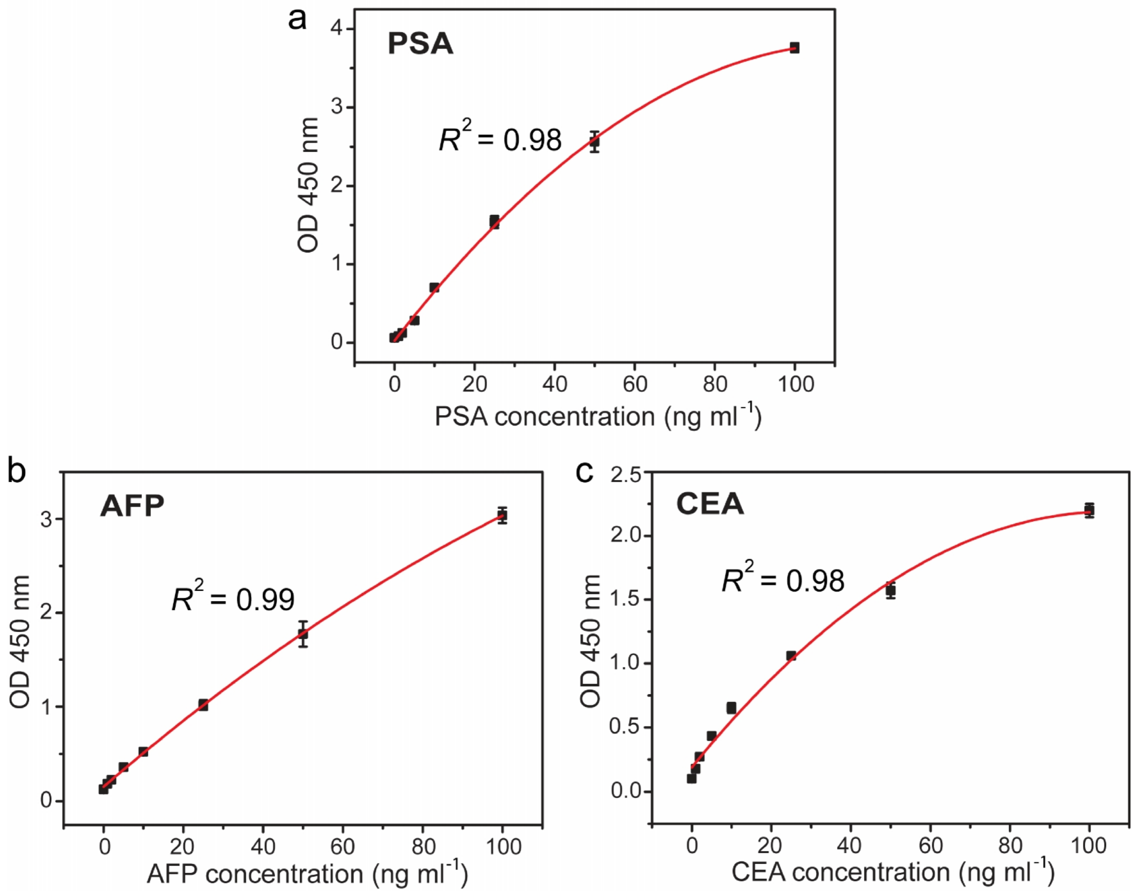


**Supplementary Figure 20.** Dose-response calibration curves for PSA (**a**), AFP (**b**) and CEA (**c**) in serum based on commercial ELISA kits. Optical density at 450 nm as a function of the biomarker concentration (ranging from 0 to 100 ng ml$^{-1}$). Samples were prepared by spiking the biomarkers in human serum albumin (HSA) solution (5%, w:w). The LOD is estimated to be 1 ng ml$^{-1}$ for PSA, AFP and CEA assessed from 3 times s.d. of blank signal. The error bar stands for s.d. of three replicates.

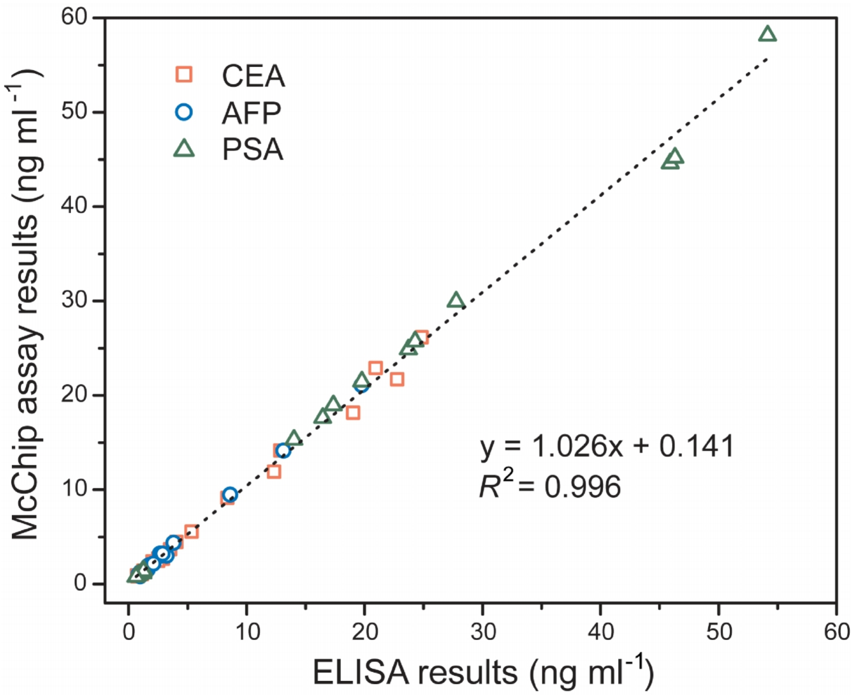


**Supplementary Figure 21.** Correlation between data obtained from the MiChip assay and ELISA for the concentrations of PSA, AFP, and CEA in 20 serum samples from cancer patients. Results show an excellent linear correlation ($R^2$ = 0.996) between the values measured by the two methods.

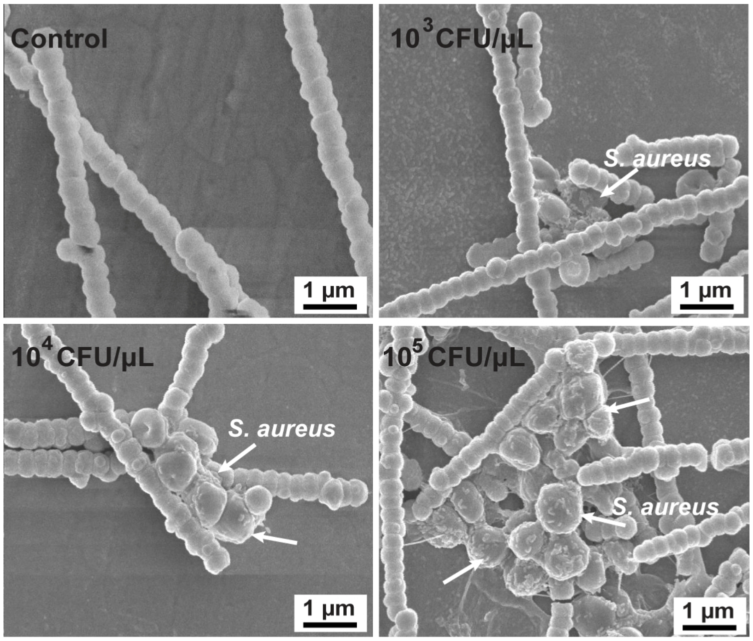


**Supplementary Figure 22.** SEM images of immune sandwich complex (Magchain - *S. aureus* - SERS probes) at varying *S. aureus* concentrations.

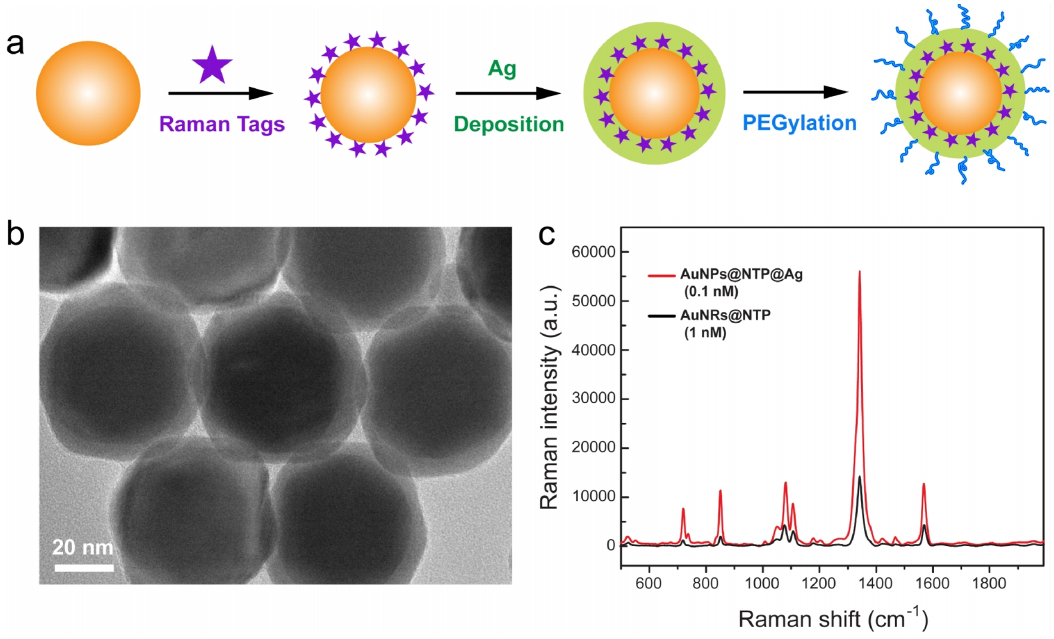


**Supplementary Figure 23.** a, Schematic illustration of the synthesis of Raman tags (*i.e.*, 4-nitrothiolphenol (NTP)) embedded SERS probes. b, TEM images of AuNPs@NTP@Ag. c, SERS spectra of AuNRs@NTP (1 nM) and AuNPs@NTP@Ag (0.1 nM).

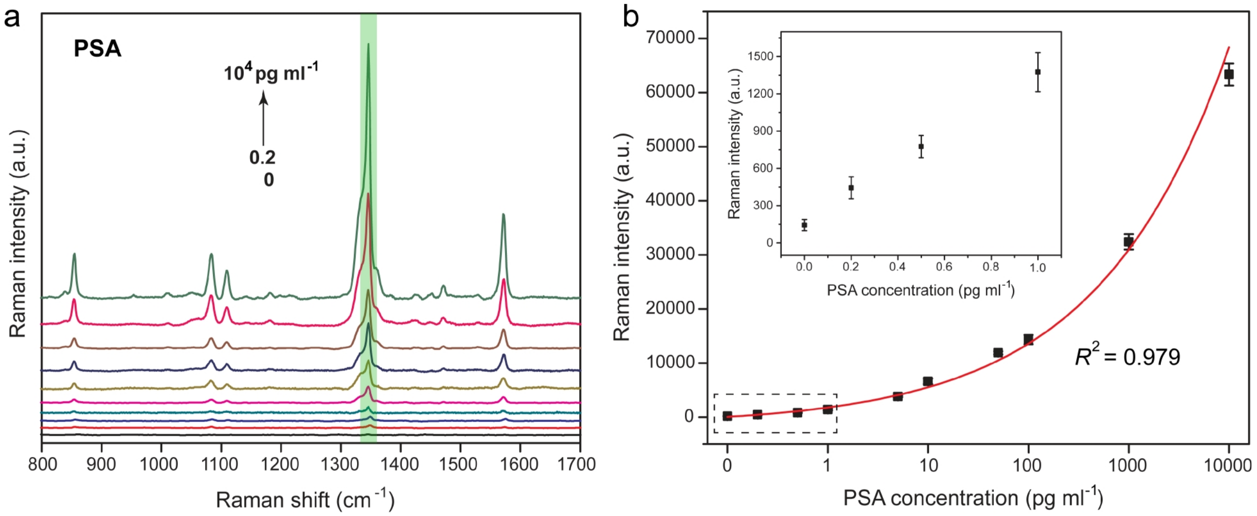


**Supplementary Figure 24. a**, SERS spectra of different concentrations of PSA detection using AuNPs@NTP@Ag as the SERS probe (ranging from 0, 0.2 to $10^4$ pg $ml^{-1}$). **b**, Standard curve for the quantitative analysis were generated by plotting the SERS peak intensity at 1341$cm^{-1}$ against the concentrations of PSA. Inset: the calibration curve at a low concentration range (PSA concentration ranged from 0 to 1 pg $ml^{-1}$). The error bar stands for s.d. of three replicates.

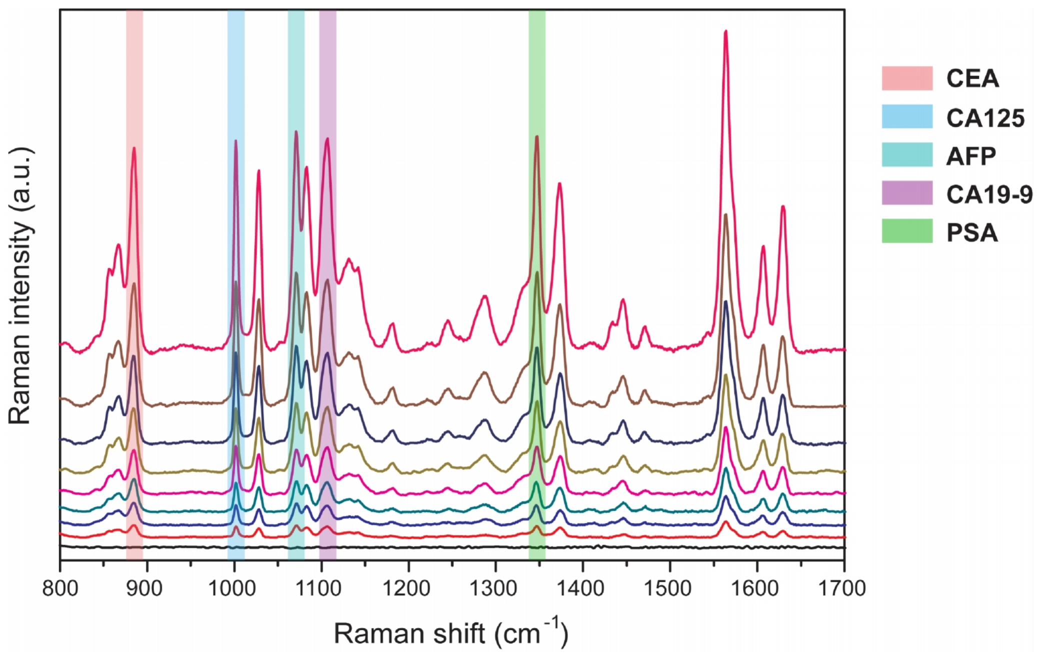


**Supplementary Figure 25**. SERS responses to a mixture of 5 cancer biomarkers (PSA: AFP: CEA= 1:1:1, ranging from 0, 0.01 to 100 ng $ml^{-1}$. CA125: CA19-9 = 1:1, ranging from 0, 0.5 to 500 U $ml^{-1}$). The Raman reporters of 4-nitrothiophenol (NTP), 4-bromothiophenol (BTP), 2,3,5,6-tetrafluorothiophenol (TFTP), 3,5-difluorothiophenol (DFTP) and 2,4-dichlorothiophenol (DCTP) correspond to the 5 cancer biomarkers PSA, AFP, CEA, CA125 and CA19-9, respectively. The well-resolved peaks at 885, 1002, 1071, 1105, and 1341 $cm^{-1}$ of the five SERS probes allow for readily simultaneous detection of their encoded targets.

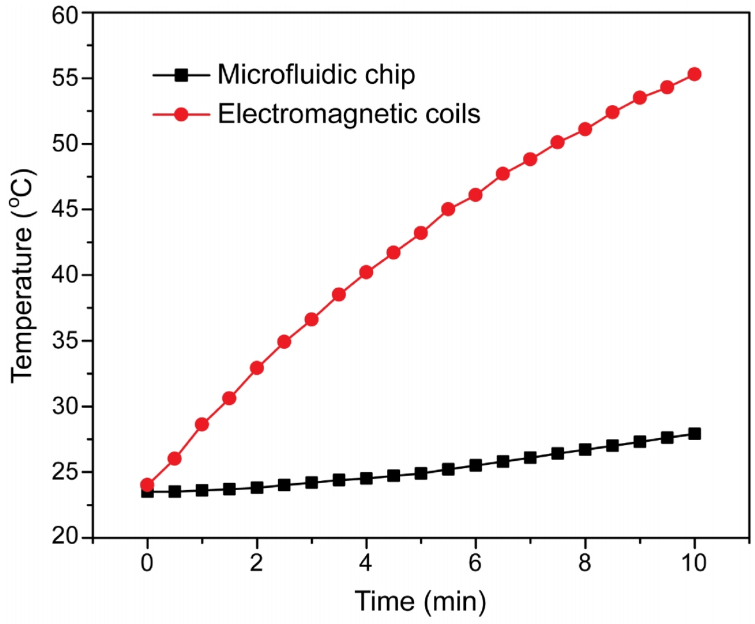


**Supplementary Figure 26.** Temperature variation of the electromagnetic coils and microfluidic chip in 10 min of operation.

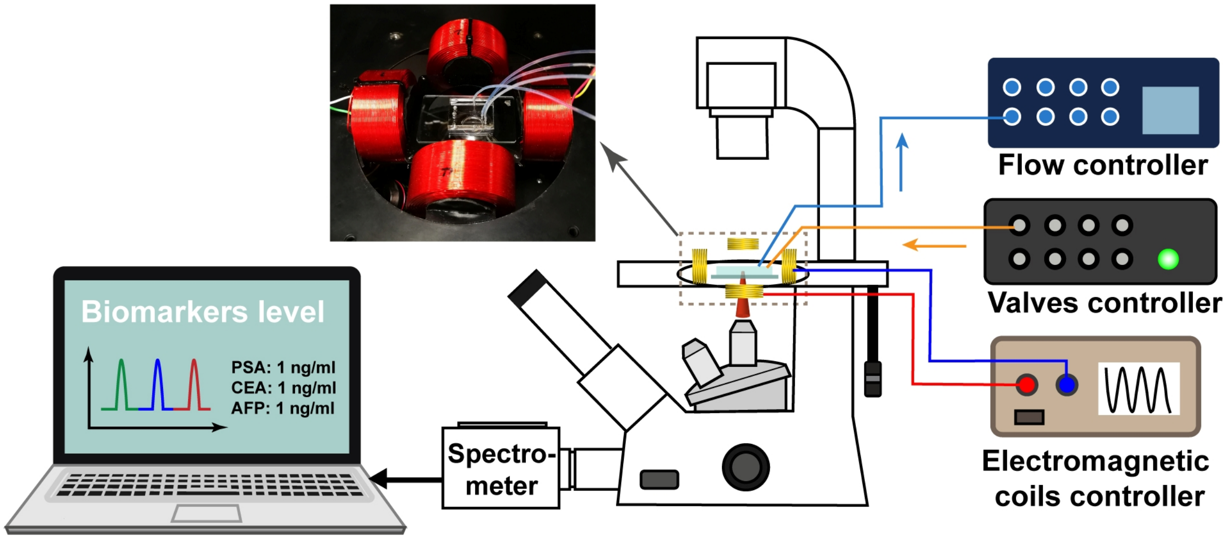


**Supplementary Figure 27.** Schematic of the MiChip assay setup including the picture of the microfluidic chip and the electromagnetic coils.

## Supplementary Tables

**Supplementary Table 1. Summary of results for 20 clinical samples from cancer patients.**

| Patients | Type of cancer* | Levels of CEA (ng ml$^{-1}$) | | Levels of AFP (ng ml$^{-1}$) | | Levels of PSA (ng m$^{-1}$) | |
|---|---|---|---|---|---|---|---|
| | | ELISA | MiChip | ELISA | MiChip | ELISA | MiChip |
| P1 | Colorectal cancer | 22.78 ± 1.45 | 21.67 ± 1.04 | 2.69 ± 0.21 | 3.18 ± 0.19 | 1.18 ± 0.12 | 1.29 ± 0.11 |
| P2 | Colorectal cancer | 12.86 ± 0.45 | 14.13 ± 0.84 | 3.83 ± 0.23 | 4.35 ± 0.43 | 0.86 ± 0.02 | 1.02 ± 0.06 |
| P3 | Colorectal cancer | 20.95 ± 1.31 | 22.89 ± 1.11 | 1.08 ± 0.05 | 0.92 ± 0.18 | 0.82 ± 0.08 | 0.97 ± 0.14 |
| P4 | Colorectal cancer | 19.03 ± 0.84 | 18.14 ± 1.29 | 1.01 ± 0.05 | 0.81 ± 0.17 | 0.91 ± 0.09 | 1.03 ± 0.11 |
| P5 | Hepatocellular carcinoma | 5.35 ± 0.27 | 5.55 ± 0.26 | 19.78 ± 0.92 | 21.07 ± 1.48 | 1.28 ± 0.03 | 1.44 ± 0.09 |
| P6 | Hepatocellular carcinoma | 2.43 ± 0.13 | 2.72 ± 0.19 | 11.62 ± 1.11 | 10.44 ± 0.93 | 1.19 ± 0.14 | 1.28 ± 0.20 |
| P7 | Colorectal cancer | 8.38 ± 0.31 | 9.14 ± 0.52 | 2.75 ± 0.16 | 2.98 ± 0.11 | 0.81 ± 0.11 | 0.94 ± 0.12 |
| P8 | Colorectal cancer | 24.86 ± 0.89 | 26.15 ± 1.28 | 1.65 ± 0.23 | 1.52 ± 0.19 | 1.23 ± 0.15 | 1.17 ± 0.13 |
| P9 | Colorectal cancer | 12.34 ± 0.69 | 11.89 ± 1.02 | 1.75 ± 0.26 | 1.95 ± 0.23 | 1.25 ± 0.01 | 1.37 ± 0.13 |
| P10 | Hepatocellular carcinoma | 4.07 ± 0.07 | 4.47 ± 0.27 | 13.15 ± 0.85 | 14.09 ± 0.54 | 0.65 ± 0.02 | 0.77 ± 0.12 |
| P11 | Prostate cancer | 1.25 ± 0.02 | 1.41 ± 0.12 | 1.15 ± 0.05 | 1.04 ± 0.11 | 14.01 ± 0.24 | 15.31 ± 0.81 |
| P12 | Prostate cancer | 1.15 ± 0.02 | 1.05 ± 0.06 | 1.02 ± 0.13 | 1.23 ± 0.15 | 45.92 ± 1.06 | 44.59 ± 2.58 |
| P13 | Prostate cancer | 3.52 ± 0.1 | 3.68 ± 0.25 | 1.55 ± 0.13 | 1.61 ± 0.09 | 23.73 ± 1.05 | 24.87 ± 1.23 |
| P14 | Prostate cancer | 2.51 ± 0.15 | 2.71 ± 0.13 | 0.87 ± 0.26 | 0.99 ± 0.14 | 24.29 ± 0.7 | 25.73 ± 0.91 |
| P15 | Prostate cancer | 3.24 ± 0.2 | 3.29 ± 0.29 | 1.34 ± 0.23 | 1.33 ± 0.18 | 27.77 ± 1.47 | 29.94 ± 0.75 |
| P16 | Prostate cancer | 2.91 ± 0.23 | 2.67 ± 0.31 | 1.67 ± 0.16 | 1.88 ± 0.22 | 17.35 ± 1.69 | 18.96 ± 1.62 |
| P17 | Prostate cancer | 2.53 ± 0.32 | 2.45 ± 0.12 | 1.23 ± 0.05 | 1.34 ± 0.21 | 46.32 ± 1.29 | 45.19 ± 1.89 |
| P18 | Prostate cancer | 0.75 ± 0.02 | 0.81 ± 0.07 | 2.12 ± 0.14 | 2.15 ± 0.27 | 16.47 ± 0.49 | 17.63 ± 1.56 |
| P19 | Prostate cancer | 0.72 ± 0.02 | 0.91 ± 0.1 | 3.26 ± 0.33 | 2.99 ± 0.29 | 54.17 ± 0.76 | 58.15 ± 1.79 |
| P20 | Prostate cancer | 2.03 ± 0.05 | 2.38 ± 0.06 | 2.9 ± 0.13 | 3.21 ± 0.28 | 19.78 ± 0.52 | 21.48 ± 1.44 |

*Cancer types were confirmed by traditional examination at Cancer Center, Union Hospital, Tongji Medical College

**Supplementary Table 2. List of antibodies used in the MiChip assay**

| Target | Function* | Vendor | Clone | Cat. # |
|---|---|---|---|---|
| Human total PSA | Ab-I | GenScript | 8A9B8 | A01664 |
| Human total PSA | Ab-II | GenScript | 1G9G8 | A01667 |
| Human CEA | Ab-I | Meridian Life Science | 057-10009 | MAM02-009 |
| Human CEA | Ab-II | Meridian Life Science | 057-10008 | MAM02-008 |
| Human AFP | Ab-I | Meridian Life Science | 131-12210 | MAM01-210 |
| Human AFP | Ab-II | Meridian Life Science | 057-11301 | MAM01-301 |
| *E. coli* O157:H7 | Ab-I | Dr. Lai Laboratory | Polyclonal Antibody | N.A. |
| *E. coli* O157:H7 | Ab-II | Dr. Lai Laboratory | 10C5-H3-B6 | N.A. |
| *S. aureus* | Ab-I | Thermo Scientific | Polyclonal Antibody | PA1-7246 |
| *S. aureus* | Ab-II | Abcam | 704 | Ab37644 |

*Ab-I: capture antibody, coupled with magnetic nanochains; Ab-II: detection antibody, conjugated with AuNRs.

## Supplementary Notes

### Supplementary Note 1: Theoretical analysis for the rotation of magnetic nanochains[1]

When an external magnetic field with magnitude $H_0$ is applied, the nanoparticles acquire an induced dipole moment $\mu$:

$$\mu = \frac{4}{3}\pi\mu_0\chi H_0 r_0^3 \quad (1)$$

where $r_0$ is the radius of the particle, $\mu_0$ is the permeability constant and $\chi$ is the magnetic susceptibility. To simplify the analysis, the effect of induced magnetic field of the paramagnetic particles on their neighbouring particles is neglected. The magnetic torque acting on a chain of $N$ units of such spherical particle is given by[2,3]:

$$\Gamma_m = \frac{3\mu^2 N^2}{64\pi\mu_0 r_0^3}\sin(2\alpha) \quad (2)$$

where $\alpha$ is the angle between the applied magnetic field and the longitudinal direction of the chain. The viscous torque which opposes the rotation of the chain is given by:

$$\Gamma_v = \kappa V\eta\omega \quad (3)$$

where $\eta$ is the viscosity of the fluid, $\omega$ is the angular frequency of the rotating magnetic field, $V$ is the volume of the magnetic chain, and $\kappa$ is the shape factor. The volume of the entire magnetic chain can be approximated as:

$$V = N\frac{4\pi(r_0 + r_c)^3}{3} \quad (4)$$

where $r_c$ is the thickness of the PDA coating which surrounds the magnetic nanoparticles to form the chain. The shape factor of a linear rigid chain formed by $N$ spherical beads is given by:

$$\kappa = \frac{2N^2}{\ln(N/2)} \quad (5)$$

A constant angular velocity is achieved when the two opposing angular torques balance each other at the center of the chain. The maximum value for $\alpha$ to maintain synchronous rotation between the chain and the applied magnetic field is $\pi/4$. Hence, the maximum applied frequency for synchronous rotation, $f_{\text{max}}$ can be found by equating Supplementary Eq 2 to Eq 3:

$$f_{\text{max}} = \frac{\chi^2 H_0^{\,2} \ln(N/2)}{16\pi N \eta \mu_0} \left( \frac{r_0}{r_0 + r_{\text{c}}} \right)^3 \quad (6)$$

In this experiment, the magnetic nanochain length ($L$) is approximated as $N \times 2r_0$. Hence, Supplementary Eq. 6 can be substituted as:

$$f_{\text{max}} = \frac{\chi^2 H_0^{\,2} r_0 \ln(L/4r_0)}{8\pi L \eta \mu_0} \left( \frac{r_0}{r_0 + r_{\text{c}}} \right)^3 \quad (7)$$

In the Supplementary Eq. 7, the terms of $\pi$ (= 3.14) and $\mu_0$ (= $1.26 \times 10^{-6}$ H m$^{-1}$) are constant. The strength of applied magnetic field ($H_0$) and the magnetic susceptibility ($\chi$) are measured to about 633.70 A m$^{-1}$ and 9.04, respectively. The viscosity of the fluid ($\eta$) is assumed to be the same as that of water ($8.90 \times 10^{-4}$ Pa.s). The radius of the magnetic nanoparticles ($r_0$) is fixed at 125 nm in this study. Hence, based on Supplementary Eq. 7, it can be determined that $f_{\text{max}}$ increases with decreasing chain length ($L$) and thickness of the PDA coating ($r_{\text{c}}$). This means that higher synchronous rotation speed can be achieved with shorter chain and thinner coating. In our experiments, the applied magnetic field frequencies are chosen according to the dimensions of the magnetic chains to ensure synchronous and steady rotation of the chain.

**Supplementary Note 2: Description on equations of mixing efficiency measurement**

The mixing efficiency between the two streams of fluid can be quantified by examining the captured fluorescent images. The intensity of the images can be correlated to the concentration of quantum dots. A MATLAB program was written to evaluate the mixing efficiency based on the changes of the fluorescent images when the two fluid streams mix. For quantifying the degree of mixing, a method based on the standard deviation of the normalized concentration profile from that of the perfectly mixed streams was adopted. The mixing efficiency, $\sigma_M$ is given by[4,5]:

$$\sigma_M = 1 - \frac{\sqrt{\frac{1}{N}\sum_{i=1}^{N}\left(\bar{c}_i - \bar{c}_\infty\right)^2}}{\sqrt{\frac{1}{N}\sum_{i=1}^{N}\left(\bar{c}_{0i} - \bar{c}_\infty\right)^2}} \quad (8)$$

where $N$ is the total number of points examined in the cross-stream direction, $\bar{c}_i$ is the normalized concentration at each point, $\bar{c}_{0i}$ is the normalized concentration at each point with no mixing taking place, and $\bar{c}_\infty$ is the normalized concentration in the complete mixing states.

The MATLAB program processed the images obtained from the fluorescent measurement by normalizing the intensity of each pixel. The concentration $c_i$ at a point for a given image can be characterized as intensity value $I_i$, and the extreme values of concentration $c_{max}$ and $c_{min}$ correspond to $I_{max}$ and $I_{min}$ respectively. The fluorescent intensity $\bar{I}_i$ can be normalized as:

$$\bar{I}_i = \frac{I_i - I_{min}}{I_{max} - I_{min}} \quad (9)$$

and the mixing efficiency may be defined as:

$$\sigma_{\mathrm{M}} = \left[ 1 - \frac{\sqrt{\frac{1}{N}\sum_{i=1}^{N}\left(\bar{I}_i - \bar{I}_\infty\right)^2}}{\sqrt{\frac{1}{N}\sum_{i=1}^{N}\left(\bar{I}_{0i} - \bar{I}_\infty\right)^2}} \right] \times 100\% \quad (10)$$

where $N$ is the total number of points examined in the cross-stream direction, $\bar{I}_i$ is the normalized intensity at each point, $\bar{I}_{0i}$ is the normalized intensity at each point with no mixing taking place (value 0 or 1), and $\bar{I}_\infty$ is the normalized intensity in the complete mixing states (value 0.5). Mixing efficiencies between the two fluids were calculated based on Supplementary Eq 10, where $\sigma_{\mathrm{M}} = 0$ indicates no mixing occurs, while $\sigma_{\mathrm{M}} = 100$ % represents complete mixing.

**Supplementary Note 3: Elimination of the black magnetic chains in image**

As shown in Supplementary Fig. 13a, the black magnetic chains in the image interfere on the evaluation of fluorescence intensity distribution. To address the problem, a permanent magnet was placed above the chamber to align the magnetic chains in the perpendicular direction so that the black magnetic chains became invisible from sight to enable accurate evaluation of the mixing efficiency (Supplementary Fig. 13b). Supplementary Fig. 13c indicates that the intensity profiles before and after the deployment of the magnet do not differ significantly. Moreover, by hiding the magnetic chains, the intensity profile is much smoother and illustrates a more accurate representation of the mixing condition in the chamber.

## Supplementary References


1 Biswal, S. L. & Gast, A. P. Rotational dynamics of semiflexible paramagnetic particle chains. *Phys. Rev. E* **69**, 041406 (2004).

2 Sandre, O. *et al.* Assembly of microscopic highly magnetic droplets: Magnetic alignment versus viscous drag. *Phys. Rev. E* **59**, 1736 (1999).

3 Wilhelm, C., Browaeys, J., Ponton, A. & Bacri, J.-C. Rotational magnetic particles microrheology: the Maxwellian case. *Phys. Rev. E* **67**, 011504 (2003).

4 Lim, C. Y., Lam, Y. C. & Yang, C. Mixing enhancement in microfluidic channel with a constriction under periodic electro-osmotic flow. *Biomicrofluidics* **4**, 014101 (2010).

5 Venancio-Marques, A., Barbaud, F. & Baigl, D. Microfluidic mixing triggered by an external LED illumination. *J. Am. Chem. Soc.* **135**, 3218-3223 (2013).
