# Supplementary material for: Magnetic nanochain integrated microfluidic biochips
Source: Nat Commun. 2018 May 1;9:1743. doi: 10.1038/s41467-018-04172-1 (PMC5931612; doi:10.1038/s41467-018-04172-1)
Supplement: Supplementary file 2 — Description of Additional Supplementary Files [file 41467_2018_4172_MOESM2_ESM.pdf]

Description of Additional Supplementary Files

File Name: Supplementary Movie 1
Description: Dark-field imaging of magnetic chains responding to an alternating magnetic field, captured by a CCD camera at 0.15 s intervals. The video shows that the magnetic nanochains immediately aligned and took synchronous, localized rotation when the spinning magnetic field was activated.

File Name: Supplementary Movie 2
Description: Measurement of the rotational speed of magnetic nanochains. High speed camera (500 frames per second) records the rotation of magnetic chains in an alternating magnetic field of different frequency.

File Name: Supplementary Movie 3
Description: Mixing enhancement by magnetic nanochains of different length (3, 10, and 20 μm), width (300, 400 and 600 nm), and concentrations (0.1, 0.25 and 0.5 mg ml-1). The video was recorded for 1 min, and was speeded up 4 times.

File Name: Supplementary Movie 4
Description: Separation of Magchains from the mixing chamber to the detection chamber. The overall duration of recording is 1.5 min. The video was speeded up 5 times.
